# Shifts in broadband power and alpha peak frequency observed during long-term isolation

**DOI:** 10.1038/s41598-020-75127-0

**Published:** 2020-10-22

**Authors:** Jan Weber, Timo Klein, Vera Abeln

**Affiliations:** 1 Institute of Movement and Neurosciences, German Sport University, Am Sportpark Muengersdorf 6, 50933 Cologne, Germany; 2 Graduate Training Center of Neuroscience, University of Tuebingen, Oesterbergstraße 3, 72074 Tuebingen, Germany; 3 Hertie-Institute for Clinical Brain Research, Otfried-Mueller-Straße 27, 72074 Tuebingen, Germany; 4 VasoActive Research Group, School of Health and Sport Sciences, University of the Sunshine Coast, Maroochydore, QLD Australia

**Keywords:** Inhibition-excitation balance, Sensory processing

## Abstract

Prolonged periods of social isolation and spatial confinement do not only represent an issue that needs to be faced by a few astronauts during space missions, but can affect all of us as recently shown during pandemic situations. The fundamental question, how the brain adapts to periods of sensory deprivation and re-adapts to normality, has only received little attention. Here, we use eyes closed and eyes open resting-state electroencephalographic (EEG) recordings to investigate how neural activity is altered during 120 days of isolation in a spatially confined, space-analogue environment. After disentangling oscillatory patterns from 1/f activity, we show that isolation leads to a reduction in broadband power and a flattening of the 1/f spectral slope. Beyond that, we observed a reduction in alpha peak frequency during isolation, but did not find strong evidence for isolation-induced changes that are of oscillatory nature. Critically, all effects reversed upon release from isolation. These findings suggest that isolation and concomitant sensory deprivation lead to an enhanced cortical deactivation which might be explained by a reduction in the mean neuronal population firing rate.

## Introduction

There is a strong consensus that oscillatory activity driven through cell assembly synchronization modulates a variety of behavioral and cognitive states, such as attention^1^, memory^2^ and sleep^3^. A vast body of literature has demonstrated the existence of several oscillatory frequency bands, ranging from approximately 0.05 Hz to 150 Hz (up to 500 Hz in animals)^4^ that have been associated with a diversity of complex cognitive operations. Furthermore, these narrowband oscillations, commonly referred to as delta (1–4 Hz), theta (4–8 Hz), alpha (8–12 Hz), beta (12–30 Hz) and gamma (> 30 Hz)^5^, have widely been studied during resting-state settings to better understand network dynamics. Multiple studies have also been employed in patient populations^6,7^ as resting-state narrowband oscillations have been shown to correlate with cognitive functions^8,9^. Therefore, resting-state measurements are extensively used to derive objective biomarkers that might be used in clinical settings^5^.

However, a ubiquitous property of such narrowband activity is its embedding within non-oscillatory, scale-free neural activity^10^ (also termed fractal component, 1/f electrophysiological noise etc.) that has until very recently just been considered as pure noise without any deeper functional meaning. Recent studies, however, revised this view and were able to demonstrate that scale-free activity might instead constitute an important behavioral and physiological index^10–13^. The 1/f component of the aperiodic signal itself is determined by the exponential decay (spectral slope) and the total offset (y-intercept) of the spectrum both of which have been shown to fluctuate with cognitive and physiological states. The spectral slope is assumed to reflect the ratio between excitation and inhibition at the synaptic level with more negative slopes reflecting enhanced inhibition^11,14^. In contrast, the spectral offset has been shown to reliably index the mean population-averaged firing rate of single units as revealed through intracranial EEG recordings^15^ as well as computational models^14^. Thus, both parameters might have the potential and robustness to serve as a proxy for neuronal population activity when only macroscopic measures are available, but information about the current cortical state wants to be inferred. It has, for example, been shown that the spectral slope reliably differentiates between different cortical states, such as sleep and wakefulness with more negative slopes during sleep indexing enhanced inhibition^16^. Another recent study revealed that medication naïve children suffering from an Attention-deficit/hyperactivity disorder (ADHD) have greater offsets and steeper slopes as compared to age-matched controls. These broadband power shifts have been proposed to be a signature of asynchronously arriving input to the dendrites of pyramidal neurons^17^. Thus, it is intelligible that broadband power increases as a function of the dendritic response to the sensory input and in turn decreases during a loss of sensory perturbation. Such a loss of sensory perturbation, commonly referred to as sensory deprivation, remains a critical point in human-isolation, both during scenarios such as COVID-19 or space flights where multisensory perception through social interaction is rare. Previous studies that looked into the effects of sensory deprivation during long-term isolation, so far, only investigated changes in oscillatory patterns, however, without accounting for potential shifts or tilts in the power spectrum. For example, previously reported decreases in alpha and beta band activity during over-wintering in the interior of Antarctica^18^ might have also originated through a decrease in broadband power or a spectral tilt towards a flatter slope during isolation when sensory input is monotonous. This is because the 1/f component of the power spectrum partially determines the oscillatory amplitude. Furthermore, as alpha peak frequency has also been shown to be a reliable predictor of cortical (de)activation that adjusts depending on the task demands^19^, caution is suggested when interpreting effects without accounting for shifts in peak frequency.

Therefore, in the present study, we took a comprehensive approach and investigated long-term-isolation-induced changes in spectral offset, slope, alpha peak frequency and oscillatory activity during both eyes opened and eyes closed resting-state conditions. To reliably estimate these features of the electrophysiological power spectrum, we used irregular resampling^20^ (IRASA) to separate oscillatory components from broadband 1/f activity. Based on aforementioned results and theoretical considerations on how the brain might adapt to sensory deprivation, we hypothesized (1) that reduced sensory input leads to reduced broadband power, (2) that spectral slopes will be steeper during isolation as a consequence of increased inhibition and less task demands and (3) that alpha peak frequency decreases during isolation as an adaptive mechanism due to reduced sensory sampling. No prior hypotheses on isolation-induced effects on oscillatory patterns were made.

## Results

An international crew of three women and three men (aged 33.67 ± 6.41 years (mean ± SD) participated in the SIRIUS-19 (Scientific International Research in Unique Terrestrial Station 2019) isolation mission and were isolated for 120 days under space analogue conditions. We recorded EEG activity at 32 scalp sites on 6 different timepoints: The first assessment day was 13 days prior to the start of the isolation period (*Pre-Isolation*). The second, third, fourth and fifth measurement were taken at isolation day 15, 54, 79 & 110, respectively. The last assessment was taken 7 days after participants were released from isolation (*Post-Isolation*). EEG was always recorded in the same settings (stationary set up in a separate laboratory compartment, noise was kept at a minimum and participants wore noise cancelling ear plugs during recordings), but recording times randomly varied between 8am and 6pm due to the complexity of international space analogue isolation missions simulating a space flight to the moon. To ascertain that the fluctuations in daytime at which we recorded EEG did not introduce any spurious effects, we performed several control analyses (see Supplementary Fig. S14). By taking recordings at multiple stages during isolation, we (1) aimed to examine if the aforementioned parameters of interest (broadband power, spectral slope and alpha peak frequency) are progressively altered as a function of time spent in isolation and (2) to ascertain that any observed change during isolation is constant across multiple stages during isolation and does not reflect a spurious fluctuation. However, as we did not observe a parametric modulation of any parameter throughout isolation (e.g. a progressive reduction of broadband power from isolation day 15 to isolation day 110), we sought to average across all isolation timepoints. This substantially reduced our degrees of freedom for follow-up post-hoc comparisons. With 6 timepoints representing 6 levels of our main factor time, we would need to perform 15 follow-up comparisons. When averaging across all isolation timepoints, the factor levels reduce from 6 to 3 and the post-hoc comparisons from 15 to 3. It is important to note, however, that the inference that can be drawn from the study does not differ if we average across isolation timepoints or treat them all separately. This can be appreciated in the Supplementary Material in Tables S1 to S5 where all possible post-hoc results for each analysis are listed.

### Sensory deprivation reduces aperiodic spectral power offset

**Figure 1:**
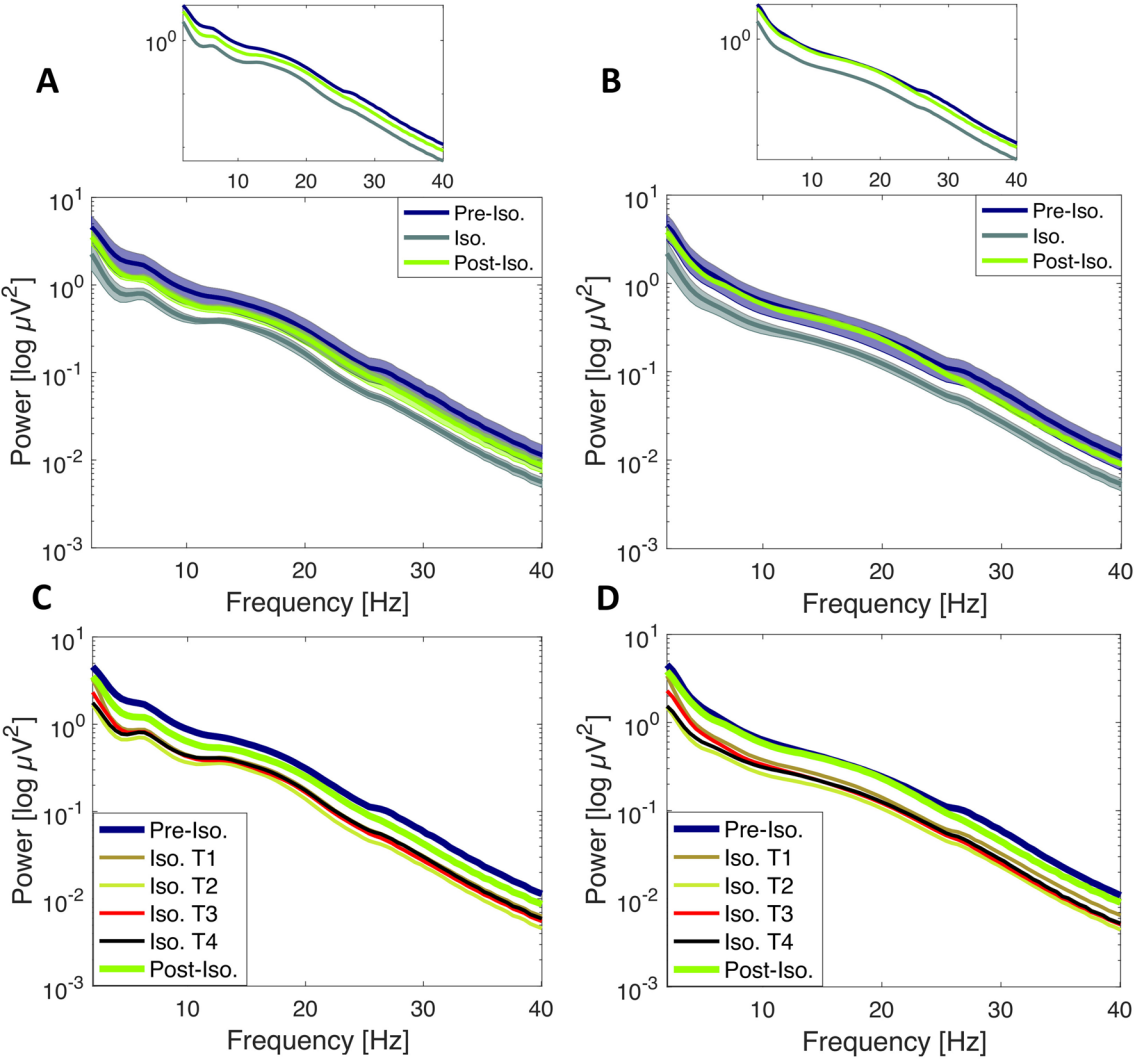
Grand average aperiodic power spectral density (PSD) after IRASA. PSD presented as mean ± SEM in semi-log power space (Figure C & D only show the mean for the purpose of visibility). (**A**) Grand average (across all participants and channels) for eyes closed condition before (*Pre-Iso.*), during (*Iso.*) and after isolation (*post-iso.*). Upper panel shows the same data without shaded error bars for visualization purposes. (**B**) Grand average (across all participants and channels) for eyes open condition (same conventions as in (**A**)). Upper panel shows the same data without shaded error bars for visualization purposes. Note the decrease in broadband power in both conditions during isolation and the rebound effect (back to baseline) post-isolation. (**C**) Grand average (across all participants and channels) for eyes closed condition during all timepoints of measurement. Note that in figure A & B, all isolation timepoints (*Iso. T1, Iso. T2, Iso. T3, Iso. T4)* were averaged together and referred to as *Iso.* (**D**) same as (**C**) only for eyes open condition. Note the consistent decrease in broadband power in both conditions during all timepoints of isolation and the rebound effect after the isolation period. *Pre Iso.* = *Pre-Isolation (− 13 days before start of isolation period), Iso. T1* =  + *15 days, Iso. T2* =  + *54 days, Iso. T3* =  + *79 days, Iso. T4* =  + *110 days, Post Iso.* = *Post-Isolation (*+ *7 days after end of isolation period)*.

In a first step we sought to test whether sensory deprivation as induced by exposure to a space-analogue environment alters the broadband power spectrum as mainly determined by the aperiodic spectral offset. We mainly predicted that broadband power would be reduced as a consequence of reduced sensory input to the brain. The grand average aperiodic power spectral density (PSD) estimates for both conditions (eyes open and eyes closed) are shown in Fig. 1. The average number of trials was 74.47 ± 3.07 and 76.42 ± 5.18 (mean ± SD) for eyes closed and eyes open, respectively. Note that for all subsequent post-hoc analyses (*spectral offset, spectral slope, alpha peak frequency, periodic PSD*), we always used the average over all data obtained during isolation and refer to this as *Iso.* (Isolation), if not stated otherwise.

**Figure 2:**
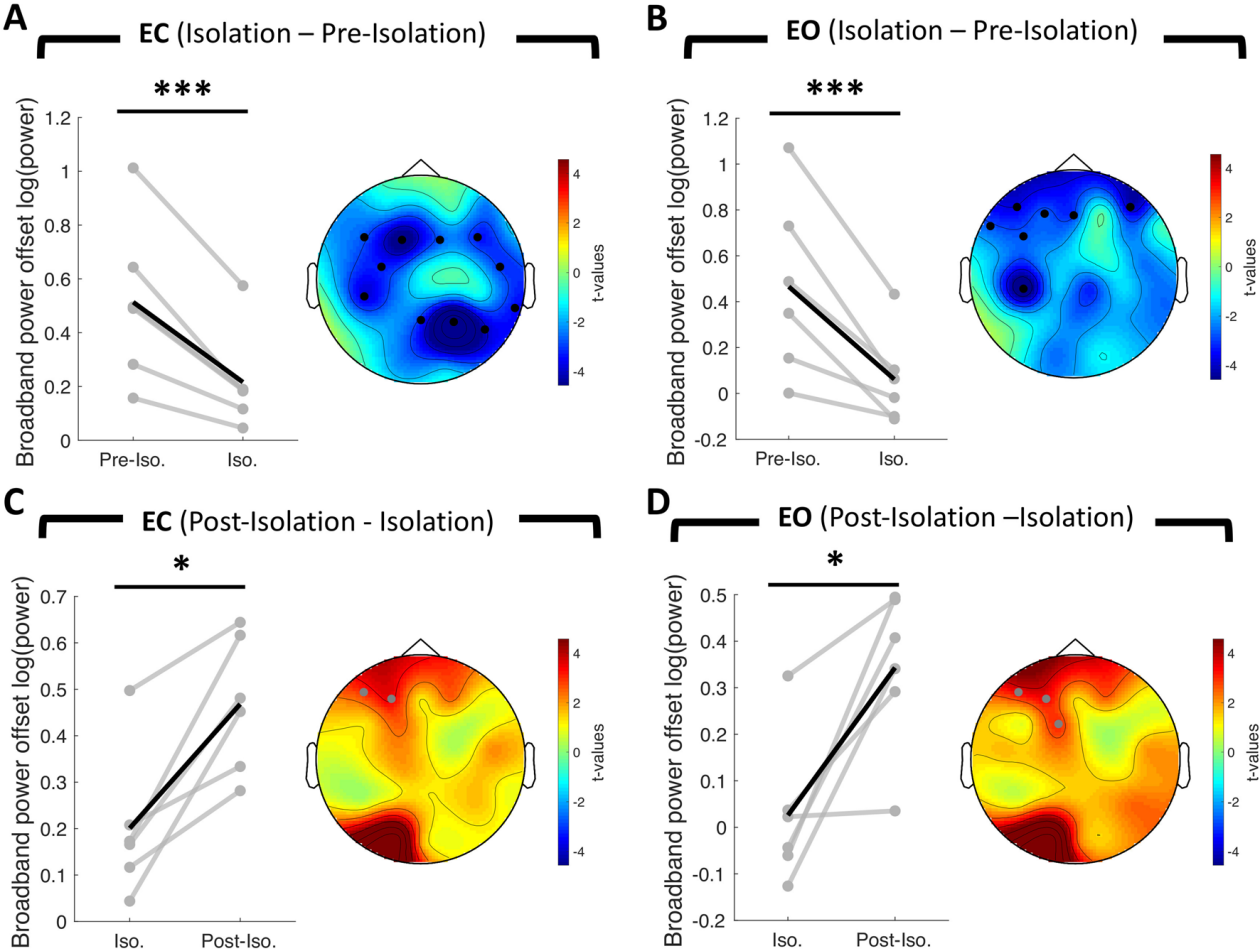
Change in spectral offset of the aperiodic signal. Gray lines represent the change in offset for individual participants (n = 6). The black line represents the mean change across participants. The topographical t-value distribution obtained via cluster permutation is plotted for each condition difference (pairwise t-tests). Black dots represent significant sensors belonging to observed negative clusters. Gray dots represent significant sensors belonging to observed positive clusters. (**A**) Change in spectral offset from pre-isolation to isolation for eyes closed condition (Cluster permutation test: n = 6, *p* < 0.001) and (**B**) eyes open condition (Cluster permutation test: n = 6, *p* < 0.001). (**C**) Change in spectral offset from isolation to post-isolation for eyes closed condition (Cluster permutation test: n = 6, *p* < 0.05) and (**D**) for eyes open condition (Cluster permutation test: n = 6, *p* < 0.05) *** *p* < 0.001, * *p* < 0.05, *EC* = *Eyes Closed*, *EO* = *Eyes Open, Pre-Iso.* = *Pre-Isolation, Iso.* = *Isolation, Post-Iso.* = *Post-Isolation*.

In order to test whether the aperiodic spectral offset was modulated across all testing sessions, we applied a within-subject repeated measures ANOVA (implemented in Fieldtrip with ft_statfun_depsamplesFunivariate), separately for eyes closed and eyes open conditions. Both conditions revealed a significant change in spectral offset over time (cluster permutation test eyes closed: *sum(F)* = 37.27, *p* < 0.01; eyes open: *sum(F)* = 24.74, *p* < 0.05). Follow-up pairwise comparisons (ft_statfun_depsamplesT) revealed a significant decrease in spectral offset upon exposure to isolation. This effect was again manifested in both resting-state conditions and was spatially extended over a broad range of electrodes (cluster permutation eyes closed: *sum(t)* = − 41.74, *p* < 0.001, *d*_*isolation* − *pre-isolation*_ = − 2.12, 11 significant channels; eyes open: *sum(t)* = − 29.39, *p* < 0.001, *d*_*isolation* − *pre-isolation*_ = − 1.88, 7 significant channels, see Fig. 2A,B).

**Figure 3:**
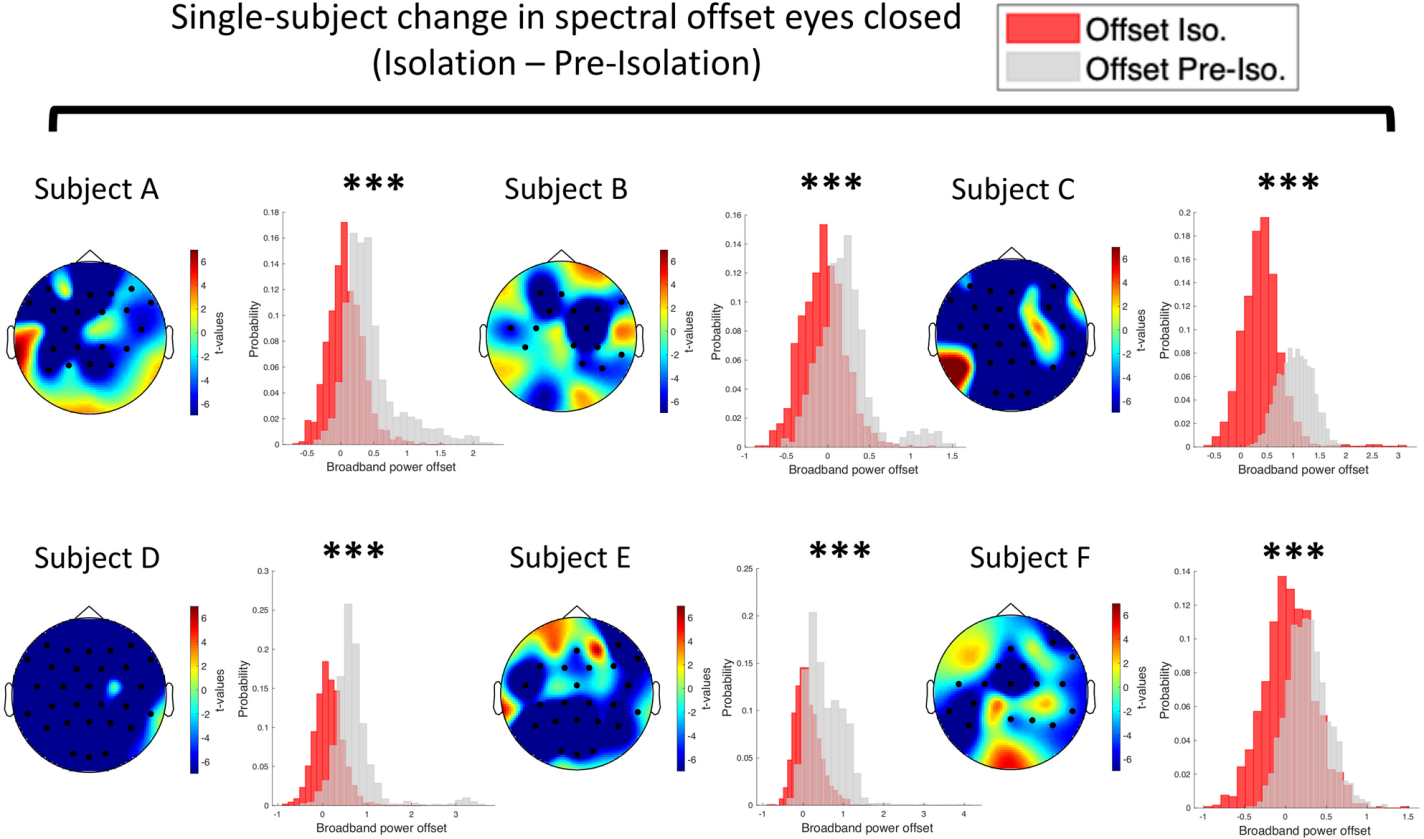
Single-subject change in spectral offset of the aperiodic signal from pre-isolation to isolation during eyes closed condition. Histograms show the probability distribution of the spectral offset. Fitting of the aperiodic signal was performed on a trial basis using IRASA. The gray histogram reflects the probability distribution of the spectral offset prior to isolation. The red histogram reflects the probability distribution of the spectral offset prior during exposure to isolation. The topographical t-value distribution (given by random trial shuffling between the two conditions, see methods for details on single-subject cluster permutation analysis) obtained via cluster permutation is plotted for each condition difference. Black dots represent significant sensors. Individual subjects are denoted as *Subject A, B, C, D, E, F*. Note the consistent global reduction (6/6 subjects show a significant decrease) in spectral power offset from pre-isolation to isolation. All other single-subject figures on spectral offset and slope differences are included in the Supplementary Material (Figs. S5–S11). ***p < 0.001.

Due to the limited number of subjects that are allowed to participate in these particular studies, we sought to further investigate the effects on a single-subject level using a cluster-based trial-shuffling procedure. Remarkably, we found robust spectral offset reductions upon exposure to isolation that were highly significant for all 6 subjects in both resting state conditions. The spatial distribution of this effect was again global (see Fig. 3 for eyes closed & Supplementary Fig. S8 for eyes open). Importantly, we observed the reversal effect in terms of spectral offset increases in both conditions once subjects were released from the space-analogue environment (cluster permutation eyes closed: *sum(t)* = 6.48, *p* < 0.05, *d*_*post-isolation* − *isolation*_ = 1.9, 2 significant channels; eyes open: *sum(t)* = 10.56, *p* < 0.05, *d*_*post-isolation* − *isolation*_ = 0.95, 3 significant channels, see Fig. 2C,D). We again examined this effect on the single-subject level and found significantly increased offsets after release from isolation in 5 out of 6 subjects in both resting-state conditions (see Supplementary Figs. S5, S9 for eyes closed and eyes open, respectively). In a final contrast, we compared the spectral offset between pre- & post-isolation, but did not find significant differences. These results indicate that sensory deprivation during isolation leads to a reduction in broadband power (here between 2 and 45 Hz) indicating an enhanced cortical deactivation. Crucially, the brain re-adapted upon release from isolation in terms of an increase in broadband power.

### Isolation-induced changes in aperiodic spectral slope

**Figure 4:**
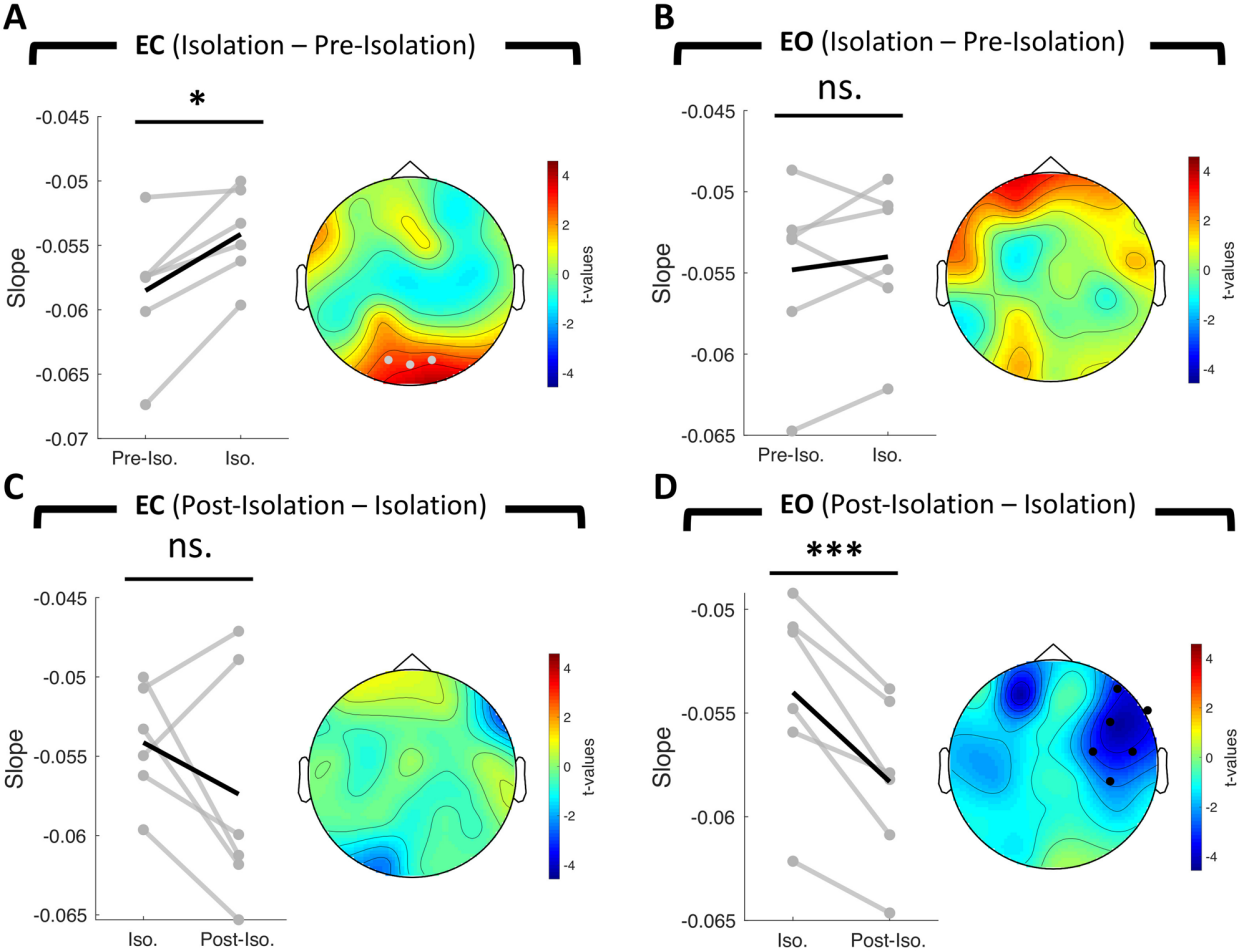
Change in spectral slope of the aperiodic signal. Gray lines represent the change in slope for individual participants (n = 6). The black line represents the mean change across participants. The topographical t-value distribution obtained via cluster permutation is plotted for each condition difference (pairwise t-tests). Black dots represent significant sensors belonging to observed negative clusters. Gray dots represent significant sensors belonging to observed positive clusters. (**A**) Change in spectral slope from pre-isolation to isolation for eyes closed condition (cluster permutation test: n = 6, *p* < 0.05) and (**B**) eyes open condition (cluster permutation test: n = 6, no resultant cluster). (**C**) Change in spectral slope from isolation to post-isolation for eyes closed condition (cluster permutation test: n = 6, no resultant cluster) and (**D**) for eyes open condition (cluster permutation test: n = 6, *p* < 0.001). ***p < 0.001, *p < 0.05, *EC* = *Eyes Closed*, *EO* = *Eyes Open, Pre-Iso.* = *Pre-Isolation, Iso.* = *Isolation, Post-Iso.* = *Post-Isolation*.

Next, we investigated whether sensory deprivation and its subsequent release modulated the aperiodic spectral slope as this has been shown to be an index for the synaptic excitatory/inhibitory balance^11,12^ with more negative slopes indexing enhanced inhibition. We essentially predicted a more negative slope during isolation as a proxy for increased cortical inhibition as a consequence of sensory deprivation. We again employed a within-subject repeated measures ANOVA, separately for both resting-state conditions, to test for a main effect of time over all sessions. However, we did not observe a significant effect over sessions (no resultant cluster). Although one should be cautious in interpreting post-hoc effects when the main effect was not significant, we performed follow up post-hoc pairwise comparisons. A group-level, non-parametric permutation approach revealed a significant increase in spectral slope from pre- to within-isolation. This effect, however, could only be observed during eyes closed resting state (*sum(t)* = 8.88, *p* < 0.05, *d*_*isolation* − *pre-isolation*_ = 1.56, 3 significant channels clustered at occipital electrodes; eyes open: no resultant cluster, see Fig. 4A,B). Although analyses on the group level are substantial for making conclusive inference, single-subject analyses are of equal importance in regards to the search of potential neurophysiological screening parameters for astronauts in space. Therefore, we again tested this effect on the single-subject domain. We found evidence for an increase in spectral slope in 4/6 subjects whereas in 2/6 we were facing the opposite pattern (in both resting-state conditions, see Supplementary Figs. S6, S10). Subsequently, we tested whether this effect reverses from within- to post-isolation. We only found evidence for a significant decrease in spectral slope after isolation for the eyes open condition (*sum(t)* = − 20.31, *p* < 0.001, *d*_*post-isolation* − *isolation*_ = − 2.13, 6 significant channels clustered at right-frontal electrodes), but not for the eyes closed condition (Fig. 4C,D). On the single-subject level, we observed spectral slope decreases post-isolation in 4/6 subjects during eyes closed and in 6/6 subjects during eyes open condition (see Supplementary Material Figs. S7, S11).

In order to test if the two main aperiodic features, spectral slope and offset are related to one another, we performed an exploratory correlation analysis and found a negative correlation between both metrics (eyes closed: two-sided, r(34) = − 0.42, *p* = 0.01, CI = [− 0.66 − 0.11]; eyes open: two-sided, r(34) = − 0.31, *p* = 0.06, CI = [− 0.58 0.02]).

### Alpha Peak Frequency is increased after release from isolation

**Figure 5:**
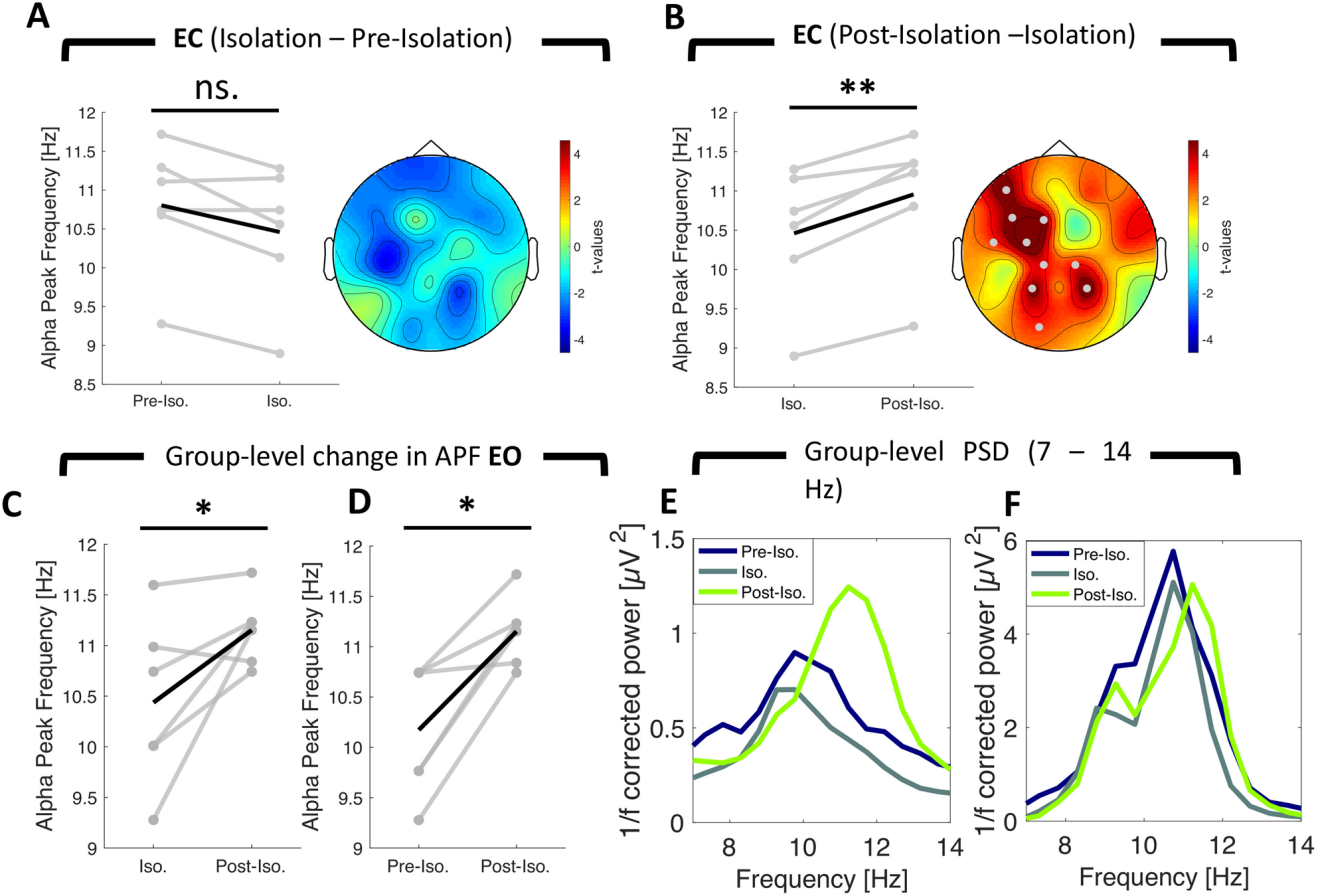
Change in Alpha Peak Frequency (APF) during eyes closed and eyes open condition after removal of the aperiodic signal using IRASA. Gray lines represent the change in APF for individual participants (n = 6). The black line represents the mean change across participants. The topographical t-value distribution obtained via cluster permutation is plotted for each condition difference (pairwise t-tests). Black dots represent significant sensors belonging to observed negative clusters. Gray dots represent significant sensors belonging to observed positive clusters. (**A**) Change in APF from pre-isolation to isolation (cluster permutation test: n = 6, *p* = 0.09) and (**B**) from isolation to post-isolation (cluster permutation test: n = 6, *p* < 0.001). (**C**) Change in APF from isolation to post-isolation (dependent t-test as detection was done on channel-averaged data: n = 6, *p* < 0.05) and (**D**) from pre-isolation to post-isolation (dependent t-test: n = 6, *p* < 0.05). (**E**) The grand average power spectral density (PSD) across all channels for the eyes open condition and (**F**) the mean 1 /f corrected PSD across all significant channels as derived via cluster-based permutation statistic. For visualization purposes, both PSDs are cut between 7 and 14 Hz. Note the increase in APF after the isolation period in both conditions. **p < 0.01, *p < 0.05, *EC* = *Eyes Closed, Pre-Iso.* = *Pre-Isolation, Iso.* = *Isolation, Post-Iso.* = *Post-Isolation*.

**Figure 6:**
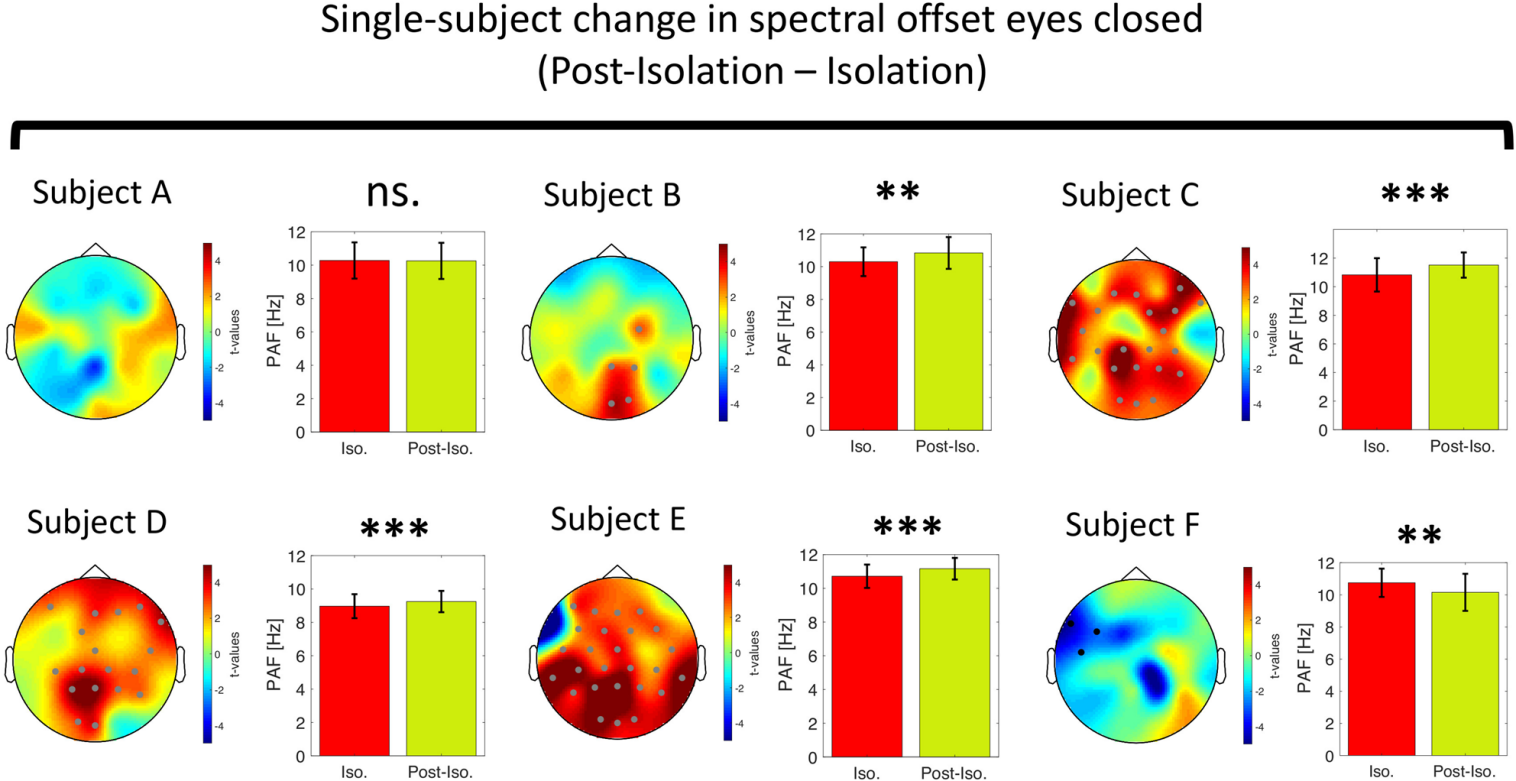
Single-subject change in Alpha Peak Frequency (APF) after removal of the aperiodic signal using IRASA. Bar plots show the mean APF during isolation (red) and post-isolation (green). Error bars represent the standard deviation over trials. The topographical t-value distribution (given by random trial shuffling between the two conditions, see methods for details on single-subject cluster permutation analysis) obtained via cluster permutation is plotted for each condition difference. Black dots represent significant sensors that are part of a negative cluster (*here:* APF increased during isolation as compared to post-isolation). Gray dots represent significant sensors that are part of a positive cluster (*here:* APF reduced during isolation as compared to post-isolation). Individual subjects are denoted as *Subject A, B, C, D, E, F*. Note that APF increased in 4/6 subjects after isolation whereas 1 subject showed the opposite pattern and in 1 subject APF was not altered. Please find the figure for single-subject comparison between pre-isolation and isolation for APF in the Supplementary Fig. S11. *p < 0.05, **p < 0.01, ***p < 0.001, *ns* not significant.

Alpha Peak Frequency (APF) has been proposed to reflect the activation level of neuronal populations with higher APF mirroring a more active and lower APF mirroring a more inactive cortical state^19^. Therefore, we predicted that APF would slow down during isolation as a consequence of reduced sensory input and increase after release from isolation as a result of re-adjustment to augmented sensory input. To test this hypothesis, we performed a detection of APF using the local maxima between 8 and 12 Hz. We performed a channel-wise peak detection (see “Methods” on how peak detection was accomplished) for the eyes closed condition as alpha peaks could be reliably estimated on a channel basis. On average only 1.06 ± 2.08 (mean ± SD) channels did not show a reliable peak. If this was the case, we interpolated the channel’s APF based on its neighbors (see “Methods” on how neighbors were defined). In contrast, a channel-averaged peak detection was performed for the eyes open condition as many channels did not show a reliable peak between 8 and 12 Hz. We used a non-parametric, cluster-based permutation approach to test for APF modulations during the eyes closed condition, whereas we employed a repeated-measures ANOVA for the eyes open condition as no inference on the spatial extent of the effect could have been made (due to the channel-averaged approach that was used). A within-subject repeated measures ANOVA revealed a significant main effect of time for both resting-state conditions (cluster permutation test eyes closed: *sum(F)* = 34.96, *p* < 0.001; repeated-measures ANOVA: *F*_*2,10*_ = 8.25, *p* < 0.01). Follow-up post-hoc testing revealed that APF increased in both conditions once subjects were released from isolation (cluster permutation eyes closed: *sum(t)* = 42.75, *p* < 0.001, *d*_*post-isolation* − *isolation*_ = 2.49, 10 significant channels [Fig. 5B]; dependent t-test eyes open: t(5) = 2.36, *p* < 0.05, *d*_*post-isolation* − *isolation*_ = 1.17 [Fig. 5C]). We again examined this effect on the single-subject level for the eyes closed condition as peak detection was reliable on a single trial level as compared to the eyes open condition (see Supplementary Fig. S3A for examples of single-trial APF estimates). Using an adaptive power threshold approach (see Methods), we discarded on average 13.99 ± 15.22% (mean ± SD) of all trials. This led to a remaining number of 60.54 ± 9.47 trials (mean ± SD) that was used for the statistical within-subject APF analysis. 4/6 subjects displayed increases in APF from within- to post-isolation whereas 1 subject displayed a decrease and 1 subject did not show any alteration (Fig. 6). We did not find strong evidence for a modulation of APF upon exposure to isolation (cluster permutation eyes closed: *sum(t)* = − 8.89, *p* = 0.09, *d*_*isolation* − *pre-isolation*_ = − 1.43, [Fig. 5A]; dependent t-test eyes open: t(5) = − 1.32, *p* = 0.12, *d*_*isolation* − *pre-isolation*_ = − 0.38), although it should be noted that there was a trend towards a decreased APF. On a single-subject level for the eyes closed condition, we observed a significant decrease in APF for 3/6 subjects. 1 subject displayed the reversed pattern and 2 subjects did not show any alteration in APF. Interestingly, we observed a significant increase in APF from pre- to post-isolation for the eyes open condition (t(5) = − 2.36, *p* < 0.05, *d*_*post-isolation* − *pre-isolation*_ = 1.97, see Fig. 5D). No such effect was found for the eyes closed condition (no resultant cluster). The oscillatory PSDs between 7 and 14 Hz for both resting state conditions are visualized in Fig. 5E,F.

**Figure 7:**
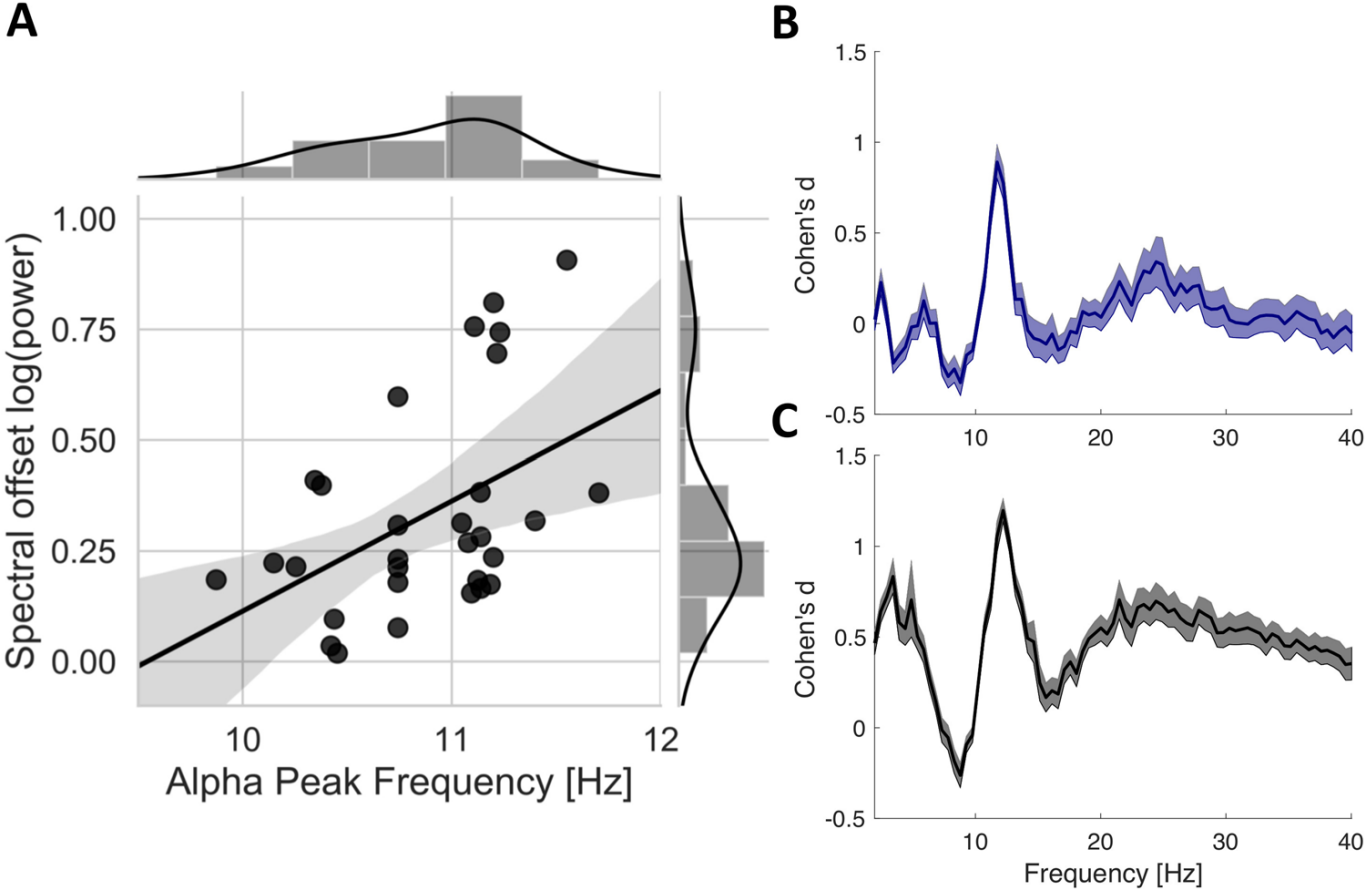
(**A**) Relationship between Alpha Peak Frequency (APF) and spectral offset of the aperiodic signal) during eyes closed condition (Spearman correlation: r(28) = 0.44, *p* < 0.01). 6 data samples (clustered around 9 Hz for APF) were removed from this correlation as they lead to a shift in the distribution of the APF data. Note, however, that the correlation is still significant even after inclusion of the 6 samples (Spearman correlation: r(34) = 0.29, *p* < 0.05). Please refer to the Supplementary Fig. S11A for scatterplots including the 6 data samples. (**B**) Channel-averaged Cohen’s d values for the periodic PSD contrast *Post-Isolation − Pre-Isolation* (Eyes Open) and (**C**) for the contrast *Post-Isolation − Isolation* (Eyes Open). Note the peak effect size in the high alpha frequency range.

Since both APF and spectral offset have been proposed as a proxy of the activation level of neuronal populations, we performed an exploratory analysis to test our prediction that both parameters should be positively correlated. We thus correlated the channel-averaged APF and spectral offset. A one-sided spearman correlation indeed revealed a significant positive correlation between both parameters for the eyes closed condition (r(28) = 0.44, *p* < 0.01, CI = [0.09 0.69], see Fig. 7A). It should be noted that for this correlation analysis, 6 data samples were excluded as they were clearly clustered away from the main data cloud and led to a non-normal distribution of the APF data. However, the inference of the correlation analysis does not change when including these data samples (Spearman correlation: r(34) = 0.29, *p* < 0.05, CI = [− 0.05 0.56], see Supplementary Fig. S13 for further explanation on why we excluded these data samples). A non-significant, but considerable trend towards a positive relationship among both parameters was found for the eyes open condition (r(34) = 0.26, *p* = 0.06, CI = [− 0.08 0.54]). These result support our initial prediction that both APF and spectral offset are related.

### No isolation-induced changes in rhythmic oscillatory activity

So far, we have demonstrated that both spectral offset and slope are significantly modulated during isolation and that APF is mainly modulated after release from isolation. In a final step, we sought to investigate overall changes in oscillatory frequency bands. We therefore subtracted the aperiodic spectral estimates that were obtained using IRASA from the spectral estimates that were obtained by regular spectral analysis, finally leading to a PSD matrix that is mostly driven by oscillatory activity and less confounded by the 1/f power law (see Supplementary Material Fig. S2 for visualization of the periodic PSD). A within-subject repeated measures ANOVA did not reveal a significant effect for the eyes closed condition (no resultant cluster). Further, upon exploratory post-hoc tests comparing pre-isolation, isolation and post-isolation separately, we did not find any change in oscillatory brain activity. However, we observed a significant modulation of oscillatory brain activity over time for the eyes open condition (*sum(F)* = 389.04, *p* < 0.01). Post-hoc pairwise comparisons revealed a significant increase in the alpha frequency range from pre- to post-isolation (*sum(t)* = 32.95, *p* < 0.001, 13 significant channels, see Fig. 7B). A similar, yet not significant pattern, could be observed when contrasting the periodic PSD during isolation and post-isolation, such that alpha power was increased post-isolation (see Fig. 7C). It should, however, be noted that by careful inspection of the periodic PSD, it becomes apparent that the change in higher alpha frequency power is mainly due to the shift in the APF, although the amplitude itself is also increased as depicted in the Supplementary Material Fig. S2B right panel.

## Discussion

The large majority of studies has particularly emphasized the role of narrowband oscillations in neuronal communication, cognition and behavior^21–24^. However, in addition to narrowband oscillations, the 1/f component of the electrophysiological power spectrum has been shown to equally index important physiological features^13,15,25^. Here, we provide first unique insights into how long-term isolation alters the electrophysiological power spectrum. We show that long-term isolation (120 days), where sensory input is at a minimum state, does not introduce strong changes in terms of narrowband (de)synchronization, but instead leads to broadband power shifts, spectral tilts as well as shifts in alpha peak frequency (APF).

### Broadband power and APF are reduced during long-term isolation

Broadband power was estimated after subtraction of band-limited oscillations using irregular resampling^20^ (IRASA) and refers to the intercept of the corrected, aperiodic-like power spectrum. We observed a reduction in broadband power (2–45 Hz) upon exposure to isolation. A rebound effect, in terms of increased broadband power, was observed upon release from isolation. Single-subject analyses using a cluster-based trial-shuffling procedure further substantiated the robustness of this observation as it was evident and consistent across all subjects in both resting-state conditions. Previous research has tried to examine the association between broadband spectral changes and activation levels of neuronal populations. One example is a study by Manning, et al.^15^ where they simultaneously recorded LFP and single-unit activity in neurosurgical patients and demonstrated that broadband LFP power across all frequencies reliably tracked single neuron activity in widespread subcortical and cortical areas. This observation has been further substantiated by Ray and Maunsell^26^ who showed a robust relationship between broadband gamma and spiking activity in macaque visual cortex. Furthermore, broadband power shifts in fusiform and parahippocampal gyri reliably classified the visual presentation of either faces or houses with high temporal precision indicating that broadband power closely tracks the activation level at local cortical circuits^17^. Even though EEG and LFP recordings both reflect the superposition of multiple transmembrane currents in the brain (e.g. synaptic activity, action potentials etc.), they do represent underlying activity at fairly different spatiotemporal scales where activity recorded at a single EEG electrode reflects a spatiotemporally smoothed version of the LFP^27,28^ Therefore, we cannot unambiguously extrapolate our findings to the aforementioned relation of broadband power to single unit activity. However, it is plausible to argue that periods of sensory deprivation and spatial confinement during long-term isolation reduce the level of vigilance and arousal which in turn might lead to an enhanced cortical deactivation. This notion can be further accentuated when taking the global, yet not significant (*p* = 0.09), reduction in APF during and its subsequent (significant) increase after isolation into consideration. The interpretation of the reduction in APF during isolation as a corollary of a reduced vigilance state can be further supported by a recent study^29^ which revealed a systematic decrease of APF over the course of an experimental session (1 h). In addition, Samaha and Postle^30^ measured the temporal resolution of visual perception by estimating the temporal threshold at which two successive light flashes could no longer be discriminated and correlated this behavioral measure with eyes-closed APF. Intriguingly, they were able to demonstrate that APF reliably tracked the temporal resolution of visual perception. That is, subjects with a higher APF had a finer temporal resolution of visual perception. Importantly, however, APF has been shown to be robust against bottom-up sensory fluctuations which suggests that APF is rather regulated in a top-down manner^31^. Our present results converge with this idea and suggest that the brain adaptively top-down regulates APF in dependence to sensory stimulation. Hence, as we show here, APF is down-regulated during isolation where sensory input is at a minimum level, but is again up-regulated post-isolation when sensory stimulation is back to normal. This interpretation is coherent with the assumption that the frequency at which active neural ensembles oscillate is self-regulated and can be described as a function of the input statistics (e.g. sensory input, task demands etc.)^19^. In order to further justify the claim that both broadband power and APF track cortical deactivation during long-term sensory deprivation, we correlated APF and broadband power, hypothesizing that if both reflect a proxy of neural de(activation), they should be positively correlated. We were indeed able to confirm our prediction by demonstrating a positive correlation between both parameters (significant for eyes closed, highly trending for eyes open). This reduction in cortical activation observed here during long-term isolation further raises the important question to what extent this effect might reflect a pathological condition. We argue that the broadband power decrease observed during isolation reflects a self-correcting mechanism where neuronal population activity is reduced as a consequence of sensory deprivation. However, in contrast to the broadband power increase observed in medication naïve young ADHD patients^13^ which might indeed reflect a disturbed feedback mechanism, we do not think that the decrease within- and subsequent increase post-isolation are signs of pathological conditions, but rather reveal a homeostatic adaption to the brain in response to reduced needs of sensory integration. We recently observed a comparable homeostatic self-regulating process for cortisol secretion during short-term isolation with highly increased cortisol values during isolation that, however, returned back to baseline values post-isolation^32^.

### Flatter 1/f spectral slope during isolation

An additional parameter that characterizes the aperiodic power spectrum is its slope. Previous work has shown that the spectral slope reflects an index of the local balance between excitation and inhibition^11,16,33^. Thereby, steeper slopes index enhanced inhibition and vice versa. Therefore, we essentially predicted that isolation would lead towards a steeper slope due to reduced excitation and relatively more inhibition as induced by a lack of sensory perturbation. However, our results point towards the opposite direction. We show that the slope increases upon exposure to isolation, however, only during the eyes closed condition. In contrast, we observed a steeper slope upon release from isolation that was significant for the eyes open condition, but the mean difference pointed towards the same direction for the eyes closed condition. This observation was also highly consistent across participants when single-subject analyses were performed. Although we expected the change in slope to point towards the opposite direction, the following scenario according to the neural noise hypothesis^12^ can be derived: The neural noise hypothesis states that increases in asynchronous and periodically decoupled population spiking leads to a flattening of the power spectrum. This feature has been shown to increase as a function of age^12^ and to mediate age-related working memory deteriorations. Therefore, it is reasonable to assume that increases in desynchronized neuronal spiking activity contributed to the flattening of the power spectrum during isolation.

### Potential mechanisms that drive spectral changes during long-term isolation

What are the potential mechanisms that drive such cortical deactivation (reduced broadband power and APF) and increases in neural noise (flatter spectral slope) during long-term isolation? Vigilance and arousal levels are known to be partially modulated by the reticular activation system (RAS) through diffuse neuromodulation^34^. Furthermore, stimulation of the RAS led to a disruption in narrowband oscillations and induced stronger desynchronized neuronal activity, prompting its potential role in regulating scale-free cortical activity^35^. This leads to the hypothesis that the ascending sensory input from the RAS to subcortical and cortical regions is reduced during periods of sensory deprivation and spatial confinement. However, further research is needed to directly assess this hypothesis. Whereas the general cortical activation state might be regulated by the ascending RAS, the change in neural noise, as measured by the slope of the aperiodic-like power spectrum, is likely to have different origins. Noradrenaline (NA) is thought to modulate sensory gain control mechanisms that regulate the balance between excitation and inhibition and determine the sensitivity of a behavioral response towards relevant sensory input^36,37^. Thus, it would be reasonable to speculate that NA and the 1/f neural noise are interrelated. A recent study^38^ could indeed provide evidence that NA as measured reliably by the pupil diameter^39^ and the 1/f neural noise are correlated. Counterintuitively, however, they showed a positive correlation suggesting that higher levels of NA indicate a flatter power spectrum and stronger neural noise. To what extent NA is altered during long-term isolation should be focus of future investigations, but initial evidence suggests that levels of NA are highly reduced during social isolation as investigated in rats^40^.

### Relation to previous isolation studies

Over the past years, a considerable number of studies has been employed to aid our understanding on how prolonged periods of isolation impact physiological mechanisms in both human and animal models^18,32,41–43^. We here address how our present results converge with previous investigations. Early studies in animal models have shown that isolation modulates the behavioral response towards novel stimuli and induces locomotor hyperactivity^44^ and leads depletions of NA in diverse brain regions including the hippocampus^45^. Furthermore, sensory deprivation as induced through isolation adversely affects cognition in animals^46^. Although the direct mechanisms underlying deterioration in cognitive performance has not been fully elucidated, it is tempting to speculate that NA might be a driving mechanism due to its important role in the induction of long-term potentiation (LTP) to facilitate memory consolidation^47^. To what extent a reduction in NA release through decreased activation of the Locus Coeruleus (LC) can explain our present observations in regards to reductions in broadband power and a flattening of the aperiodic slope remains to be seen. However, it has been indeed demonstrated that LC stimulation leads to a stronger rhythmicity in spike timing and a more reliable response towards external stimuli^47^ which would well align with the hypothesis that lower levels of NA decrease arousal and cortical activation. So far, the effect of isolation on human physiology has only been studied on a macroscopic level. However, our results align well with previous studies showing a reduction in global brain activity in the alpha and beta frequency during isolation^18,42,43^. Yet, it should be noted once more that in comparison to previous studies that only focused on changes in oscillatory activity over the course of isolation, we here applied an approach to disentangle oscillatory and non-oscillatory (aperiodic) features. Thereby, we showed that isolation induces a reduction in broadband power rather than changes in narrowband oscillations. Therefore, we suggest that future studies investigating changes in neural activity during isolation follow this approach to prevent potential conflation of changes in rhythmic and arrhythmic activity.

## Conclusion

In conclusion, we have demonstrated that both broadband power and APF are reduced during and subsequently increased after isolation. This suggests that long-term isolation reduces cortical activation levels. As previous studies have found a strong correlation between broadband power across all frequencies and single-unit activity, our findings let us to speculate that the mean population firing rate is reduced during long-term isolation. Furthermore, our data reveals a trend towards a flatter spectral slopes during isolation indicating more desynchronized spiking activity. In conjunction, our results are the first ones to show that isolation induces changes in (1) broadband EEG power, (2) alpha peak frequency and (3) 1/f neural noise. It is, however, important to note that, although isolation studies provide unique insights into how long-term isolation shapes cortical activity, we cannot simply extrapolate our findings onto effects of pandemic situations and concomitant isolation. This is in particular because the circumstances, such as financial security, general health (not being exposed to a risk situation through a virus) and the concern regarding the health status of other family members, remain stationary during isolation studies whereas they are uncertain and highly unpredictable during pandemic situations.

## Limitations

We address three major limitations of the present study.

(1) We did not include a control group with which potential interaction effects could have been studied to highlight that our observations were indeed caused by isolation. However, we were able to show in previous studies that a non-isolated control group did not show any alterations over time^32^. Thus, we believe that our results here are caused by sensory deprivation during long-term isolation. (2) Due to the high logistic expense and cost effort due to the nature of these particular studies that try to mimic a space-analogue conditions, the sample size is extremely low. However, we tried to statistically analyze the data in a way (non-parametric cluster-based permutations and single-subject analyses) that allows us inferring the conclusions that we finally made.

(3) Some confounding factors, that might explain some variance in our results, such as mood, boredom or shifts in sleep cycles, cannot be entirely excluded. We do, however, believe that these factors are not orthogonal to the phenomenon of sensory deprivation, but rather describe psychological consequences of prolonged periods of monotony and sensory deprivation.

## Materials and methods

### Participants

The study was approved by the ethic committee of the Institute of Biomedical Problems (IMBP) as well as the Institutional Review Board of the National Aeronautics and Space Administration (NASA). An international crew of three women and three men (aged 33.67 ± 6.41 years (mean ± SD) participated in the SIRIUS-19 (Scientific International Research in Unique Terrestrial Station 2019) isolation mission. All methods were performed in accordance with the relevant guidelines and regulations and informed consent was obtained from all participants.

### Experimental procedure

The mission was conducted in the NEK (Nezemnyy Eksperimental´nyy Kompleks) multicompartment facility of the IMBP at the Russian Academy of Sciences (RAS) in Moscow under the leadership of the IMBP and NASA Human Research Program and participation of the German (DLR) and French (CNES) Space Agencies. The crew was isolated for 120 days under space analogue conditions from March to July 2019 simulating a journey to the Moon with extravehicular activities on the lunar surface and back to Earth. Detailed information about the SIRIUS-19 mission scenario can be found at: https://www.nasa.gov/sites/default/files/atoms/files/sirius_19_booklet.pdf. Besides the medical and laboratory compartment, in which the present experimental recordings were realized, NEK facilities include separated compartments for physical training, kitchen and living room, private dorms, storage, lavatories and technical operations.

### Data acquisition timepoints

We recorded EEG activity at 32 scalp sites on 6 different timepoints: The first assessment day was 13 days prior to the start of the isolation period (*Pre-Isolation*). The second, third, fourth and fifth measurement were taken at isolation day 15, 54, 79 and 110, respectively. The last assessment was taken 7 days after participants were released from isolation (*Post-Isolation*). EEG was always recorded in the same settings (stationary set up in a separate laboratory compartment, noise was kept at a minimum and participants wore noise cancelling ear plugs during recordings), but recording times randomly varied between 8am and 6 pm due to the complexity of internal space analogue isolation missions simulating a space flight to the moon. To ascertain that the fluctuations in daytime at which we recorded EEG did not introduce any spurious effects, we performed several control analyses (see Supplementary Fig. S14).

### EEG data acquisition

EEG data was obtained during eyes closed and eyes open resting state conditions while subjects were seated in a relaxed position. Both conditions were continuously recorded for 2.5 min with a transition period of 30 s.

EEG data was recorded at a sampling rate of 1000 Hz using an electrode cap Ag/AgCl active electrodes (ActiCap EEG Active Electrode System, Brain Products GmbH, Gilching, Germany) located at 32 scalp sites based on an international 10–20 system^48^.

### EEG preprocessing

Preprocessing and statistical analysis of the data was carried out in MATLAB 2019a (MathWorks Inc.) using custom written code along with functions from the Fieldtrip toolbox^49^. EEG data analysis was performed separately for the eyes open and eyes closed condition. All analyses steps, however, were equal for both conditions. In order to prevent edge artifacts, we applied a bandpass-filter between 1 and 45 Hz to the continuous data. Data was then demeaned, detrended and re-referenced the data to an average reference before we separated the data into non-overlapping epochs of 2 s. Subsequently, data was visually inspected and trials containing visually prominent artifacts were rejected. Subsequently, we used an independent-component analysis (ICA) to remove eye-blinks, saccades or cardiac artefacts. We then extracted the variance of the signal channel-wise for each trial by averaging over channels. Trials were rejected if the variance exceeded the 1.5 times the interquartile range of the median variance. In a final step, we again visually inspected the signal for residual artifacts and rejected artifact-contaminated trials.

### Spectral decomposition

We computed the spectral estimates by means of a Fast-Fourier Transformation after applying a single Hanning window to all artefact-free segments. Spectral estimates were obtained between 2 and 45 Hz in steps of 0.5 Hz on single trials. For later group-level analyses, we averaged the power spectrum along the trial dimension. We limited the analysis to 45 Hz to prevent confounds in later analyses on spectral slope and broadband power estimates due to line noise at 50 Hz. Besides, no further additional information was expected in the higher frequency ranges. To reliably estimate offset, slope and alpha peak frequency (APF) of the electrophysiological power spectrum, we used irregular resampling^20^ (IRASA) to separate oscillatory components from broadband 1/f contribution. To extract purely oscillatory features from the signal, we subtracted the spectral estimates that were obtained using IRASA from the spectral estimates that were obtained by regular spectral analysis^50^. This procedure allowed us to estimate the APF by means of the purely oscillatory driven signal as well as spectral slope and broadband power (offset of the spectral signal) by means of the aperiodic 1/f signal that itself has been shown to encompass information about the physiological state^25^.

### Spectral slope and offset estimation

We estimated spectral slope and spectral offset on the aperiodic signal using IRASA. The key aperiodic features (slope and offset) were calculated by fitting a linear regression to the aperiodic signal in semi-log power space (*polyfit.m,* MATLAB and Curve Fitting Toolbox Release R2015a, The MathWorks, Inc., Natick, Massachusetts, United States)^13^. Fitting was performed channel-wise on the trial-averaged data for group-level analysis and on a trial-basis for single-subject analysis.

### Analysis of Alpha Peak Frequency

We calculated the Alpha Peak Frequency (APF) by means of the periodic signal that entails the residual oscillatory components of the signal. This approach facilitates the identification of a spectral peak and prevents misleading detection of a peak that is purely explained by the 1/f contribution to the signal^51,52^. As a reminder, the purely oscillatory driven signal was obtained by subtracting the aperiodic 1/f contribution from the original power spectrum. We used a semi-automatic detection for APF detection. We predefined a range for APF between 8 and 12 Hz. The algorithm then extracted the APF that was defined as the local power maxima between 8 and 12 Hz. To finally determine whether the local maximum indeed reflected a peak, we used an adaptive power threshold procedure. Therefore, we first computed the power spectrum for each channel and derived its mean and standard deviation over the whole frequency range. We then multiplied the standard deviation by a factor of 3 and added the resulting product to the spectral mean. This computation finally resulted in a single number that predefined the threshold for a peak to be considered as robust and reliable for further statistical analysis. We visually inspected the power spectrum and confirmed the decision of the algorithm on each trial. There was, however, no need for manual adjustment. APF detection was performed channel-wise on the trial-averaged data for group-level analysis and on a trial-basis for single-subject analysis.

### Statistical analysis

We used cluster-based permutation tests to test for changes in spectral slope, offset and APF over time^53^. Since, to the best of our knowledge, no prior study has investigated the effects of isolation and sensory deprivation on spectral slope, offset or APF, no prior assumptions on the spatial extent of the effects were made. Therefore, we decided against testing the effects on a certain subset of electrodes, but to take advantage of the non-parametric cluster-based permutation approach that provides insights into the spatial extent of the effect while still correcting for the multiple comparison problem. Statistical effects were employed both on the group as well as on the single-subject level. We used exactly the same statistical approach for examining changes in spectral slope, offset and APF.

#### Group-level analysis

In a first step, we employed a non-parametric, cluster-based within-subject repeated measures ANOVA (implemented in Fieldtrip with ft_statfun_depsamplesFunivariate) to assess the main effect of time using all timepoints (6 factor levels: pre-isolation, isolation day 15, 54, 79, 110, post-isolation). To ascertain that the main effect of time was still present when we averaged across all isolation timepoints (isolation day 15, 54, 79 and 110), we also employed the ANOVA on the isolation-averaged data (3 factor levels: pre-isolation, average isolation, post-isolation). This extra ANOVA was conducted as post-hoc tests were employed on the isolation-averaged data (see beginning of results section for a more detailed explanation).

Follow-up pairwise comparisons were performed using cluster-based permutation tests. Permutation distributions for higher group level analysis were obtained by randomly shuffling the condition labels (timepoints of interest, e.g. *pre-isolation vs. isolation*). Due to the small sample size, we used all possible random partitions (here: 6^2^ = 64) to derive the permutation distribution. For every random partition, we contrasted the two conditions of interest using dependent-sample t-tests on the channel-level. Spatial clusters were formed by thresholding at the t-statistic at a critical alpha of *0.05*. Clusters were formed by two or more neighboring channels in space. The selection of neighbors was based on the triangulation method. The maximum t-statistic within a spatial cluster was calculated by summation of all t-values. Finally, the largest t-sum among all clusters was extracted. Repeating this step for the maximal number of possible permutations ensured a distribution of the maximum t-statistics. The p-value was then computed by calculating the proportion of random partitions that exceeded the observed (true) test statistic. The p-value was directly adjusted for two-tailed testing and thereby multiplied by a factor of two.

#### Single-subject analysis

We followed the same rationale to test for effects on the single-subject level. However, instead of randomly shuffling the condition labels on the subject-level, we randomly permuted the labels on a trial-level. That is, the mean value of a single channel was derived by averaging across trials instead of averaging across subjects.

## Supplementary information

Supplementary Information.

## Data Availability

The datasets generated during and/or analyzed during the current study are available from the corresponding author upon reasonable request.
