# Supplementary material for: Shifts in broadband power and alpha peak frequency observed during long-term isolation
Source: Sci Rep. 2020 Oct 22;10:17987. doi: 10.1038/s41598-020-75127-0 (PMC7581825; doi:10.1038/s41598-020-75127-0)
Supplement: Supplementary file 1 — Supplementary Information. [file 41598_2020_75127_MOESM1_ESM.pdf]

Supplementary Information

Shifts in broadband power and alpha peak frequency observed during long-term isolation

Jan Weber [1,2,3*], Timo Klein [1,4], Vera Abeln [1*]

[1] Institute of Movement and Neurosciences, German Sport University, Am Sportpark Muengersdorf 6, 50933 Cologne, Germany

[2] Graduate Training Center of Neuroscience, University of Tuebingen, Oesterbergstraße 3, 72074 Tuebingen, Germany

[3] Hertie-Institute for Clinical Brain Research, Otfried-Mueller-Straße 27, 72074 Tuebingen, Germany

[4] VasoActive Research Group, School of Health and Sport Sciences, University of the Sunshine Coast, Maroochydore DC, QLD, Australia.

* Corresponding authors

Contact Info

Jan Weber, Institute of Movement and Neurosciences, German Sport University, Am Sportpark Muengersdorf 6, 50933 Cologne, Germany.

Email: jan.weber@student.uni-tuebingen.de

Timo Klein, Institute of Movement and Neurosciences, German Sport University, Am Sportpark Muengersdorf 6, 50933 Cologne, Germany.

Email: t.klein@dshs-koeln.de

Phone: +49 221 4982 4260

Vera Abeln, Institute of Movement and Neurosciences, German Sport University, Am Sportpark Muengersdorf 6, 50933 Cologne, Germany.

Email: v.abeln@dshs-koeln.de

Phone: +49 221 4982 4210

Table S1: Complete post-hoc comparisons for power offset eyes closed condition (cluster-based permutation tests, Monte-Carlo method, 1000 iterations)

Power offset eyes closed

Session	*Pre Iso.*	*T2*	*T3*	*T4*	*T5*	*Post Iso.*
Pre Iso.	-	< 0.001 ↓	< 0.001 ↓	< 0.001 ↓	< 0.001 ↓	ns.
T2	-	-	ns.	< 0.001 ↓	ns.	ns.
T3	-	-	-	ns.	ns.	ns.
T4	-	-	-	-	ns.	ns.
T5	-	-	-	-	-	< 0.05 ↑
Post Iso.	-	-	-	-	-	-

↓ = Decrease in offset ↑ = Increase in offset
Pre Iso. = Pre-Isolation (-13 days before start of isolation period), *T2* = +15 days, *T3* = +54 days, *T4* = +79 days, *T5* = + 110 days, *Post Iso.* = Post-Isolation (+ 7 days after end of isolation period)

Table S2: Complete post-hoc comparisons for power offset eyes open condition (cluster-based permutation tests, Monte-Carlo method, 1000 iterations)

Power offset eyes open

MD	*Pre Iso.*	*T2*	*T3*	*T4*	*T5*	*Post Iso.*
Pre Iso.	-	< 0.001 ↓	< 0.001 ↓	< 0.05 ↓	< 0.05 ↓	ns.
T2	-	-	ns.	ns.	ns.	ns.
T3	-	-	-	ns.	ns.	< 0.001 ↑
T4	-	-	-	-	ns.	ns.
T5	-	-	-	-	-	< 0.001 ↑
Post Iso.	-	-	-	-	-	-

↓ = Decrease in offset ↑ = Increase in offset
Pre Iso. = Pre-Isolation (-13 days before start of isolation period), *T2* = +15 days, *T3* = +54 days, *T4* = +79 days, *T5* = + 110 days, *Post Iso.* = Post-Isolation (+ 7 days after end of isolation period)

Table S3: Complete post-hoc comparisons for spectral slope eyes closed condition (cluster-based permutation tests, Monte-Carlo method, 1000 iterations)

Spectral slope eyes closed

MD	*Pre Iso.*	*T2*	*T3*	*T4*	*T5*	*Post Iso.*
Pre Iso.	-	ns.	ns.	ns.	ns.	ns.
T2	-	-	< 0.05 ↑	ns.	ns.	ns.
T3	-	-	-	ns.	ns.	ns.
T4	-	-	-	-	ns.	ns.
T5	-	-	-	-	-	ns.
Post Iso.	-	-	-	-	-	-

↓ = Decrease in spectral slope ↑ = Increase in spectral slope
Pre Iso. = Pre-Isolation (-13 days before start of isolation period), *T2* = +15 days, *T3* = +54 days, *T4* = +79 days, *T5* = + 110 days, *Post Iso.* = Post-Isolation (+ 7 days after end of isolation period)

Table S4: Complete post-hoc comparisons for spectral slope eyes open condition (cluster-based permutation tests, Monte-Carlo method, 1000 iterations)

Spectral slope eyes open

MD	*Pre Iso.*	*T2*	*T3*	*T4*	*T5*	*Post Iso.*
Pre Iso.	-	ns.	ns.	ns.	ns.	< 0.05 ↓
T2	-	-	ns.	ns.	ns.	ns.
T3	-	-	-	ns.	ns.	ns.
T4	-	-	-	-	ns.	ns.
T5	-	-	-	-	-	< 0.05 ↓
Post Iso.	-	-	-	-	-	-

↓ = Decrease in spectral slope ↑ = Increase in spectral slope
Pre Iso. = Pre-Isolation (-13 days before start of isolation period), *T2* = +15 days, *T3* = +54 days, *T4* = +79 days, *T5* = + 110 days, *Post Iso.* = Post-Isolation (+ 7 days after end of isolation period)

Table S5: Complete post-hoc comparisons for Alpha Peak Frequency (APF) eyes closed condition (cluster-based permutation tests, Monte-Carlo method, 1000 iterations)

Alpha Peak Frequency eyes closed

MD	*Pre Iso.*	*T2*	*T3*	*T4*	*T5*	*Post Iso.*
Pre Iso.	-	ns.	< 0.05 ↓	< 0.001 ↓	ns.	ns.
T2	-	-	ns.	< 0.001 ↓	ns.	< 0.05 ↑
T3	-	-	-	ns.	ns.	< 0.01 ↑
T4	-	-	-	-	ns.	< 0.001 ↑
T5	-	-	-	-	-	< 0.01 ↑
Post Iso.	-	-	-	-	-	-

↓ = Decrease in APF ↑ = Increase in APF

Pre Iso. = Pre-Isolation (-13 days before start of isolation period), *T2* = +15 days, *T3* = +54 days, *T4* = +79 days, *T5* = + 110 days, *Post Iso.* = Post-Isolation (+ 7 days after end of isolation period)

Figure S1: Regular PSD including each isolation timepoint

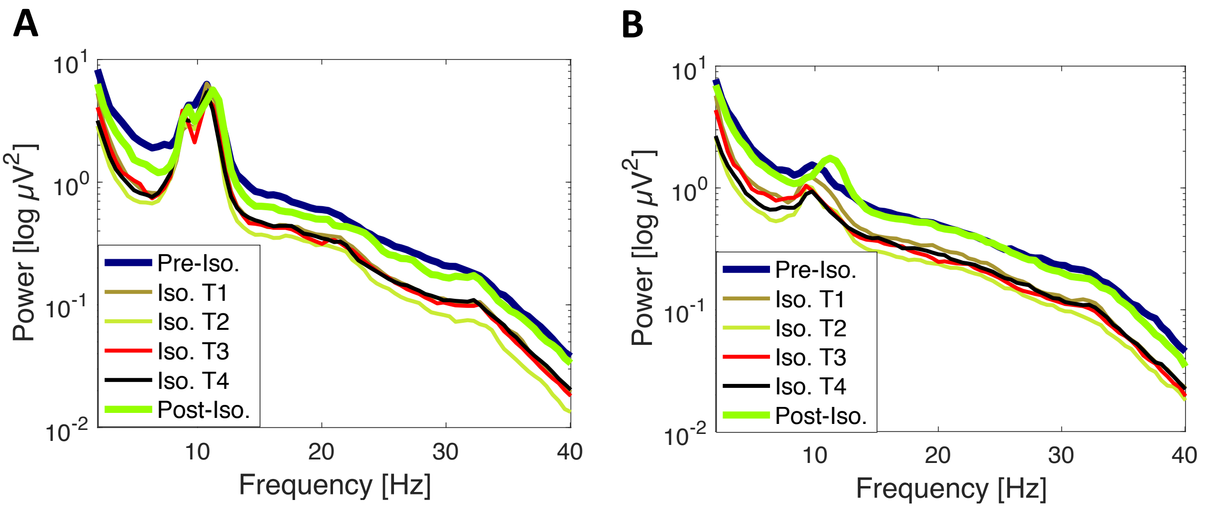

Regular PSD (oscillatory + non-oscillatory components) estimates including all timepoints represented on a semi-log power scale (A) Regular PSD (eyes closed condition) obtained from spectral decomposition before subtraction of the aperiodic part. (B) Same as (A) only for eyes open condition.

Legend: Pre Iso. = Pre-Isolation (-13 days before start of isolation period), Iso. T1 = +15 days, Iso. T2 = +54 days, Iso. T3 = +79 days, Iso. T4 = + 110 days, Post Iso. = Post-Isolation (+ 7 days after end of isolation period)

Figure S2: Periodic (oscillatory) power spectrum after extracting rhythmic features of the EEG signal via Irregular Resampling Auto-Spectral Analysis (IRASA)

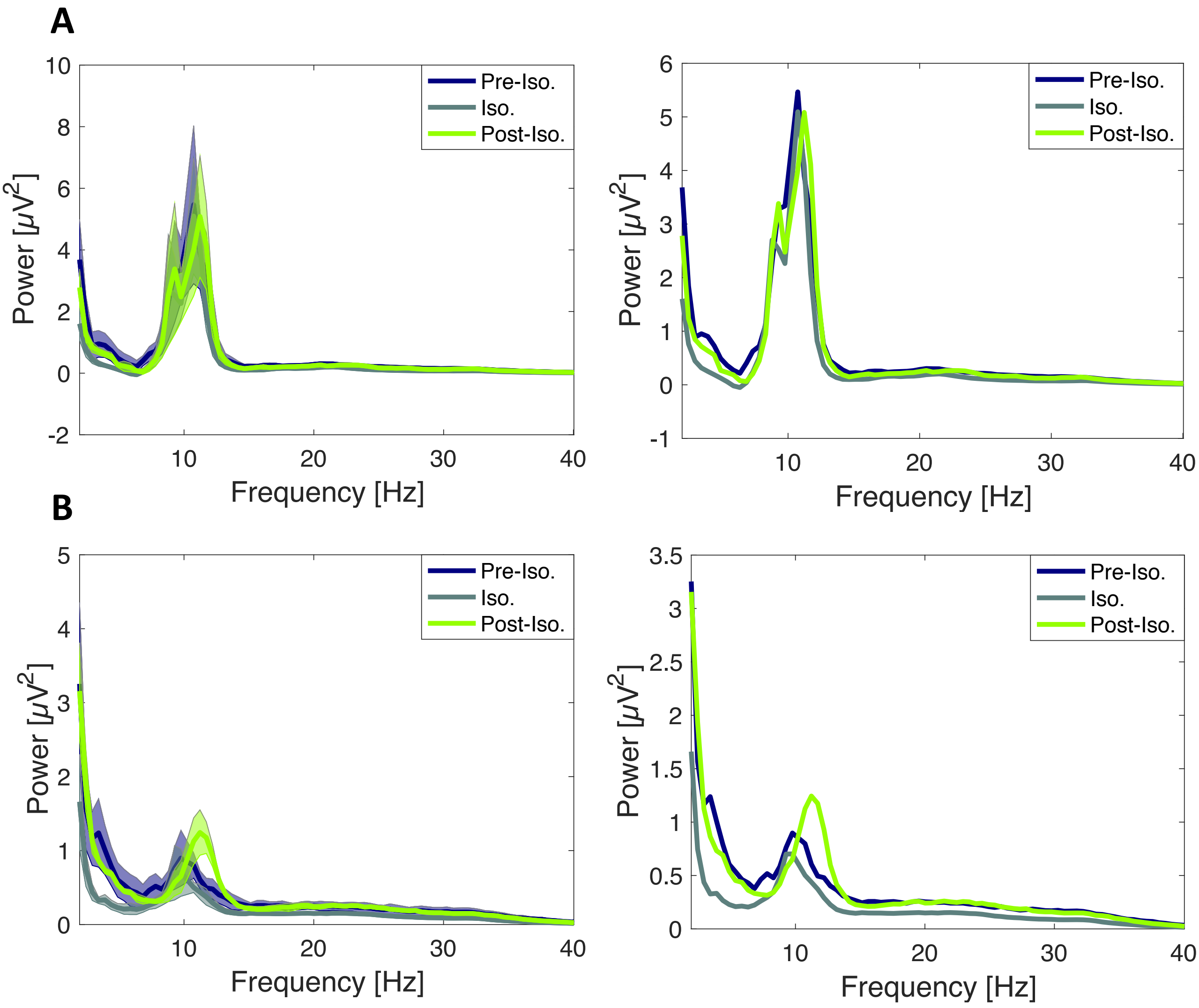

Power spectral density (PSD) between 1 – 40 Hz. (A) PSD for eyes closed after removal of aperiodic features (left panel = mean ± SEM, right panel = mean). (B) PSD for eyes open after removal of aperiodic features (left panel = mean ± SEM, right panel = mean).
Legend: Pre-Iso. = Pre-Isolation (-13 days before start of isolation period), Iso. = Isolation, Post-Iso. = Post-Isolation (+ 7 days after end of isolation period)

Figure S3: Example robustness of spectral fitting and alpha peak estimates on a single-trial level

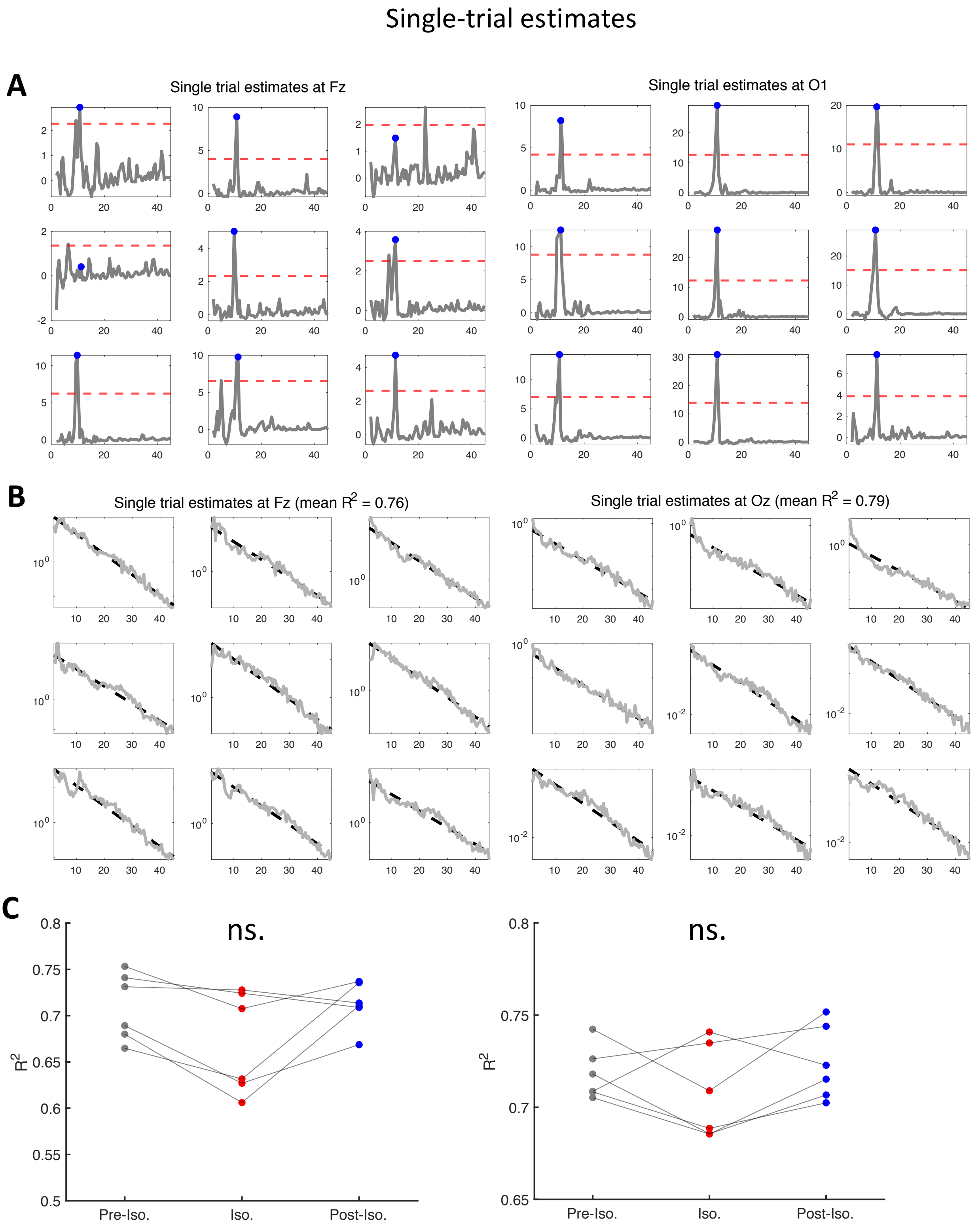

(A) Left panel: Examples of single-trial estimates of alpha peak frequency (APF) for channel *Fz*. Different subplots represent different trials. Red dashed lines represent the threshold that determined whether a peak was considered as a reliable peak (*see* Methods for how the threshold was determined). Right panel: Same conventions as in left panel, only for channel *Oz*.

(B) Left panel: Examples of single-trial fitting of the aperiodic power spectrum after IRASA for channel *Fz*. Different subplots reflect different trials. The dashed line represents the best fit via least-square regression. The gray line reflects the PSD in semi-log power space. R^2 represents the goodness of fit. Right panel: Same conventions as left panel, only for channel *Oz*.

(C) Left panel: Mean goodness of fit (R^2, averaged over channels and trials) based on single-trial fitting of the aperiodic PSD for the eyes closed condition. The dots reflect the goodness of fit for each participant. R^2 values did not differ between the timepoints of interest (pre-isolation, isolation, post-isolation; repeated-measures ANOVA: $F_{2,5} = 3.8$, $p = 0.06$). Right panel: Mean goodness of fit (R^2, averaged over channels and trials) based on single-trial fitting of the aperiodic PSD for the eyes open condition. The dots reflect the goodness of fit for each participant. R^2 values did not differ between the timepoints of interest (pre-isolation, isolation, post-isolation; repeated-measures ANOVA: $F_{2,5} = 2.02$, $p = 0.18$).

Figure S4: Example robustness of spectral fitting and alpha peak estimates on a trial-averaged level

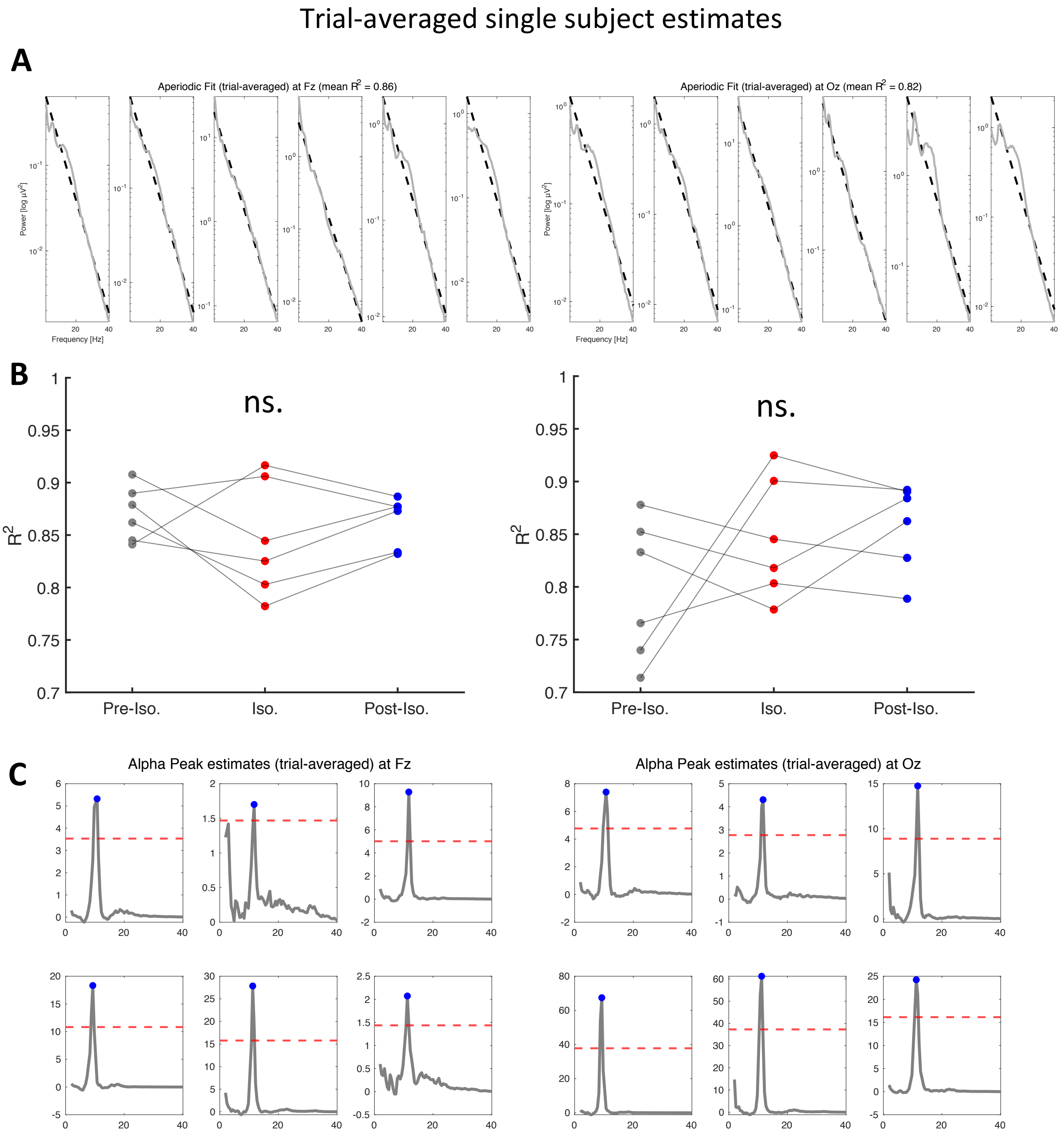

(A) Left panel: Each subplot reflects the fitting of the aperiodic PSD in semi-log power space for one individual participant at channel *Fz*. The dashed line represents the best fit via least-square regression. The gray line reflects the PSD in semi-log power space. R^2 denotes the goodness of fit. Right panel: Same conventions as in left panel, only for channel *Oz*.

(B) Left panel: Mean goodness of fit (R^2, averaged over channel) based on trial-averaged fitting of the aperiodic PSD for the eyes closed condition. The dots reflect the goodness of fit for each participant. R^2 values did not differ between the timepoints of interest

(pre-isolation, isolation, post-isolation; repeated-measures ANOVA: $F_{2,5}$ = 0.86, p = 0.45). Right panel: Mean goodness of fit (R^2, averaged over channel) based on trial-averaged fitting of the aperiodic PSD for the eyes open condition. The dots reflect the goodness of fit for each participant. R^2 values did not differ between the timepoints of interest (pre-isolation, isolation, post-isolation; repeated-measures ANOVA: $F_{2,5}$ = 1.64, p = 0.24).

(C) Left panel: Trial-averaged alpha peak estimates at channel *Fz*. Each subplot represents one participant. Red dashed lines represent the threshold that determined whether a peak was considered as a reliable peak (*see* Methods for how the threshold was determined). Right panel: Same conventions as left panel, only for channel *Oz*.

Figure S5: Change in spectral offset from isolation to post-isolation on a single-subject level for eyes closed condition

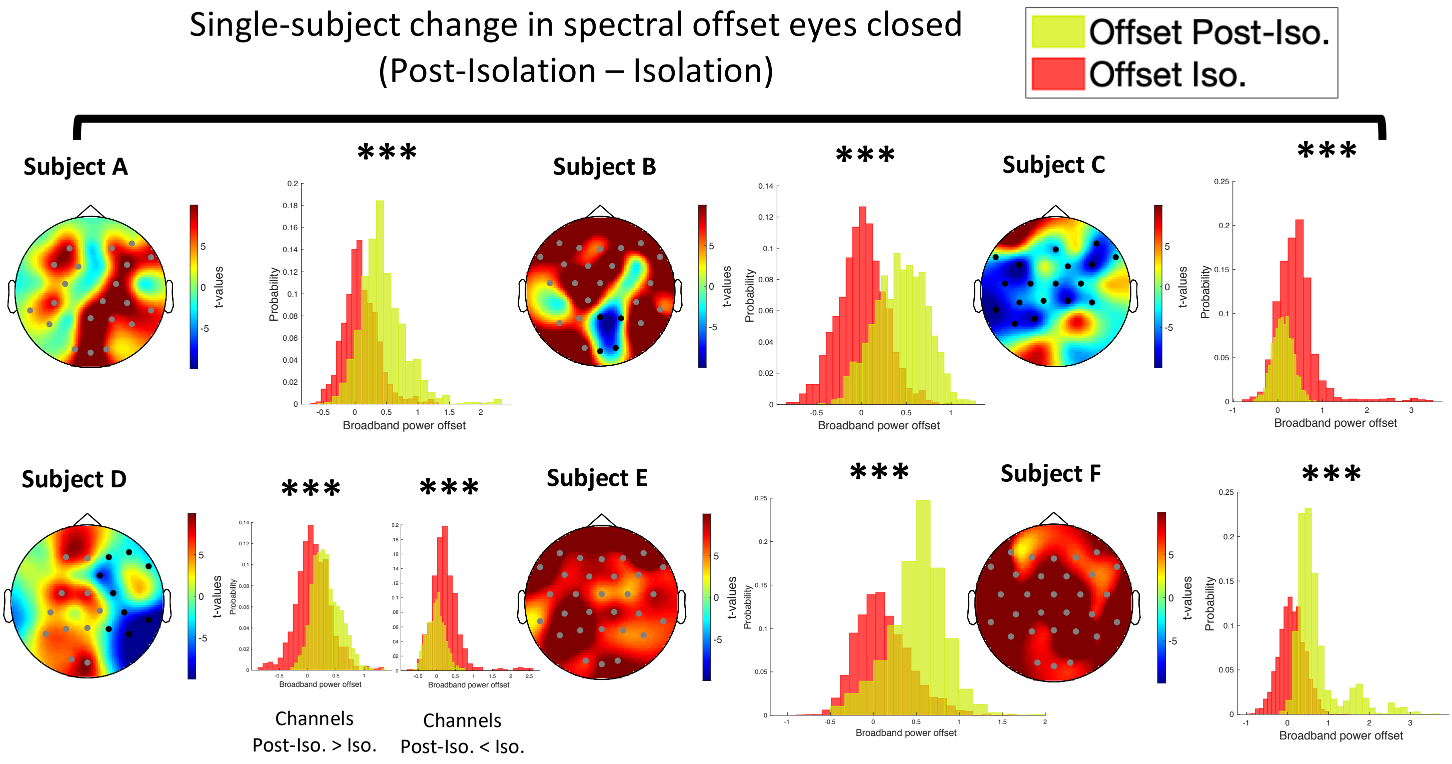

Single-subject change in spectral offset of the aperiodic signal from isolation to post-isolation during eyes closed condition. Histograms show the probability distribution of the spectral offset on a trial basis. Fitting of the aperiodic signal was performed on a trial basis using IRASA. The red histogram reflects the probability distribution of the spectral offset during isolation. The green histogram reflects the probability distribution of the spectral offset after exposure to isolation (post-isolation). The topographical t-value distribution (obtained through random trial shuffling between the two conditions, *see* methods for details on single-subject cluster permutation analysis) obtained via cluster permutation is plotted for each condition difference (pairwise t-tests). Black dots represent significant sensors that are part of a negative cluster (*here:* spectral offset higher during isolation as compared to post-isolation). Gray dots represent significant sensors that are part of a positive cluster (*here:* spectral offset lower during isolation as compared to post-isolation). Individual subjects are denoted as *Subject A, B, C, D, E, F*. Note the consistent global reduction (5/6 subjects show a significant decrease; subject D shows both increases and decreases in spectral offset which are clustered separately approximately along the two hemispheres) in spectral power offset from isolation to post-isolation.

Legend: *** $p < 0.001$, ** $p < 0.01$

Figure S6: Change in spectral slope from pre-isolation to isolation on a single-subject level for eyes closed condition

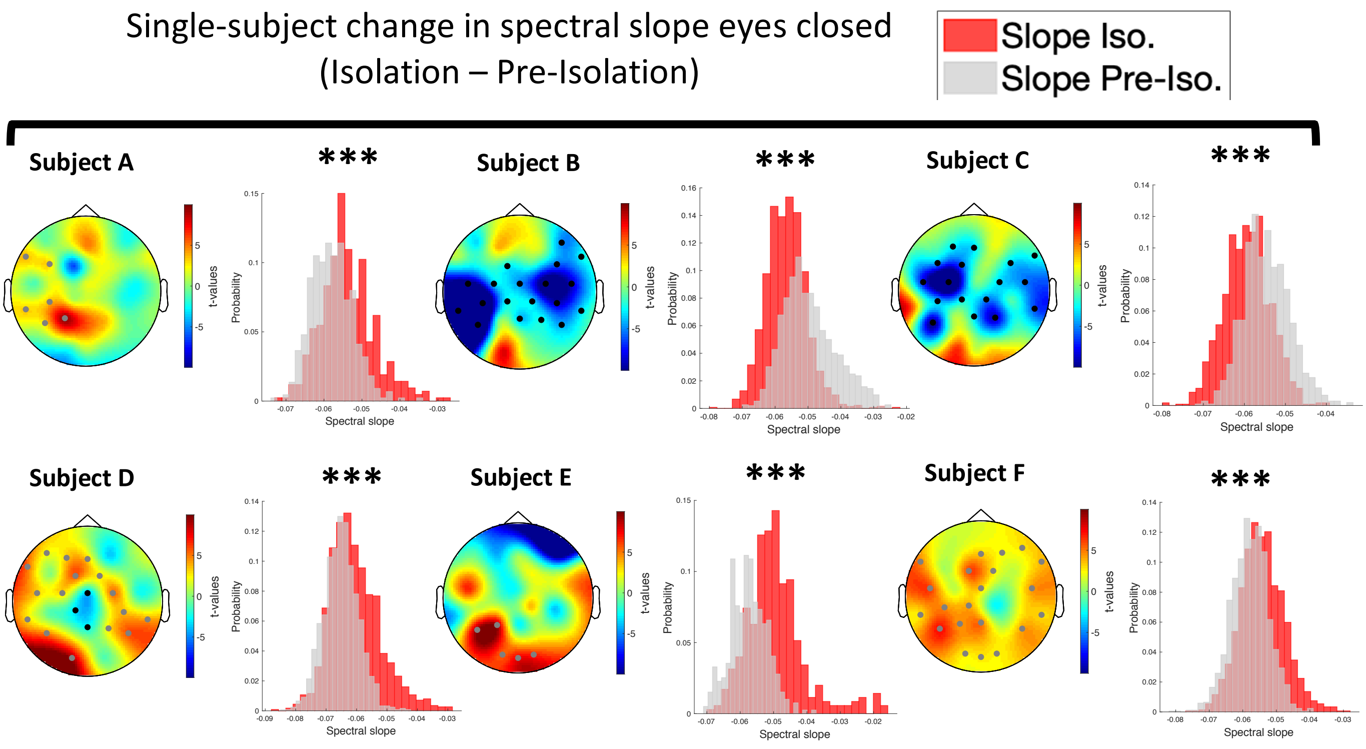

Single-subject change in spectral slope of the aperiodic signal from pre-isolation to isolation during eyes closed condition. Histograms show the probability distribution of the spectral slope on a trial basis. Fitting of the aperiodic signal was performed on a trial basis using IRASA. The gray histogram reflects the probability distribution of the spectral slope prior to isolation. The red histogram reflects the probability distribution of the spectral slope during isolation. The topographical t-value distribution (obtained through random trial shuffling between the two conditions, *see* methods for details on single-subject cluster permutation analysis) obtained via cluster permutation is plotted for each condition difference (pairwise t-tests). Black dots represent significant sensors that are part of a negative cluster (*here:* spectral slope steeper during isolation as compared to pre-isolation). Gray dots represent significant sensors that are part of a positive cluster (*here:* spectral slope flatter during isolation as compared to pre-isolation). Individual subjects are denoted as *Subject A, B, C, D, E, F*. Note that 4/6 subjects show an increase in spectral slope from pre-isolation to isolation whereas 2/6 subjects show the opposite pattern.
Legend: *** $p < 0.001$

Figure S7: Change in spectral slope from isolation to post-isolation on a single-subject level for eyes closed condition

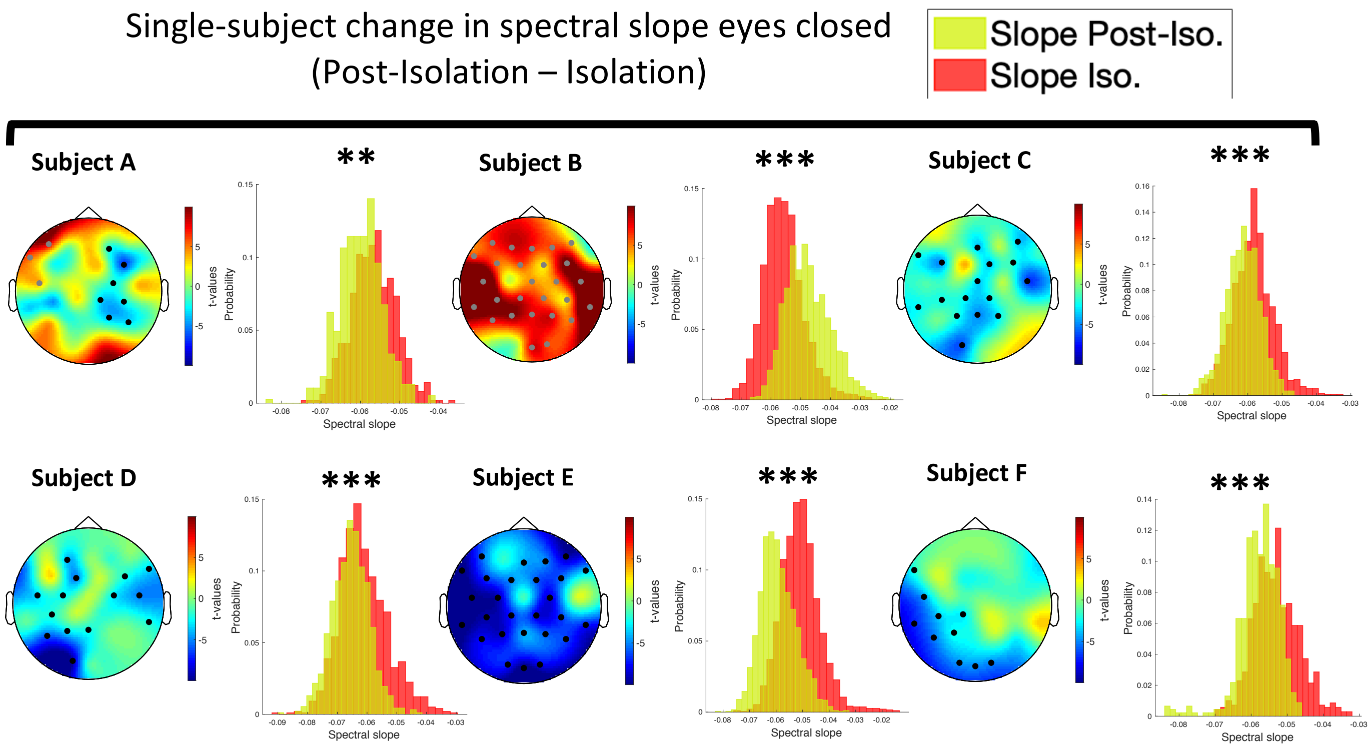

Single-subject change in spectral slope of the aperiodic signal from isolation to post-isolation during eyes closed condition. Histograms show the probability distribution of the spectral slope on a trial basis. Fitting of the aperiodic signal was performed on a trial basis using IRASA. The red histogram reflects the probability distribution of the spectral slope during isolation. The green histogram reflects the probability distribution of the spectral slope post-isolation. The topographical t-value distribution (obtained through random trial shuffling between the two conditions, *see* methods for details on single-subject cluster permutation analysis) obtained via cluster permutation is plotted for each condition difference (pairwise t-tests). Black dots represent significant sensors that are part of a negative cluster (*here:* spectral slope flatter during isolation as compared to post-isolation). Gray dots represent significant sensors that are part of a positive cluster (*here:* spectral slope steeper during isolation as compared to post-isolation). Individual subjects are denoted as *Subject A, B, C, D, E, F*. Note that 5/6 subjects show a decrease in spectral slope from isolation to post-isolation whereas 1 subject shows the opposite pattern.
Legend: *** $p < 0.001$

Figure S8: Change in spectral offset from pre-isolation to isolation on a single-subject level for eyes open condition

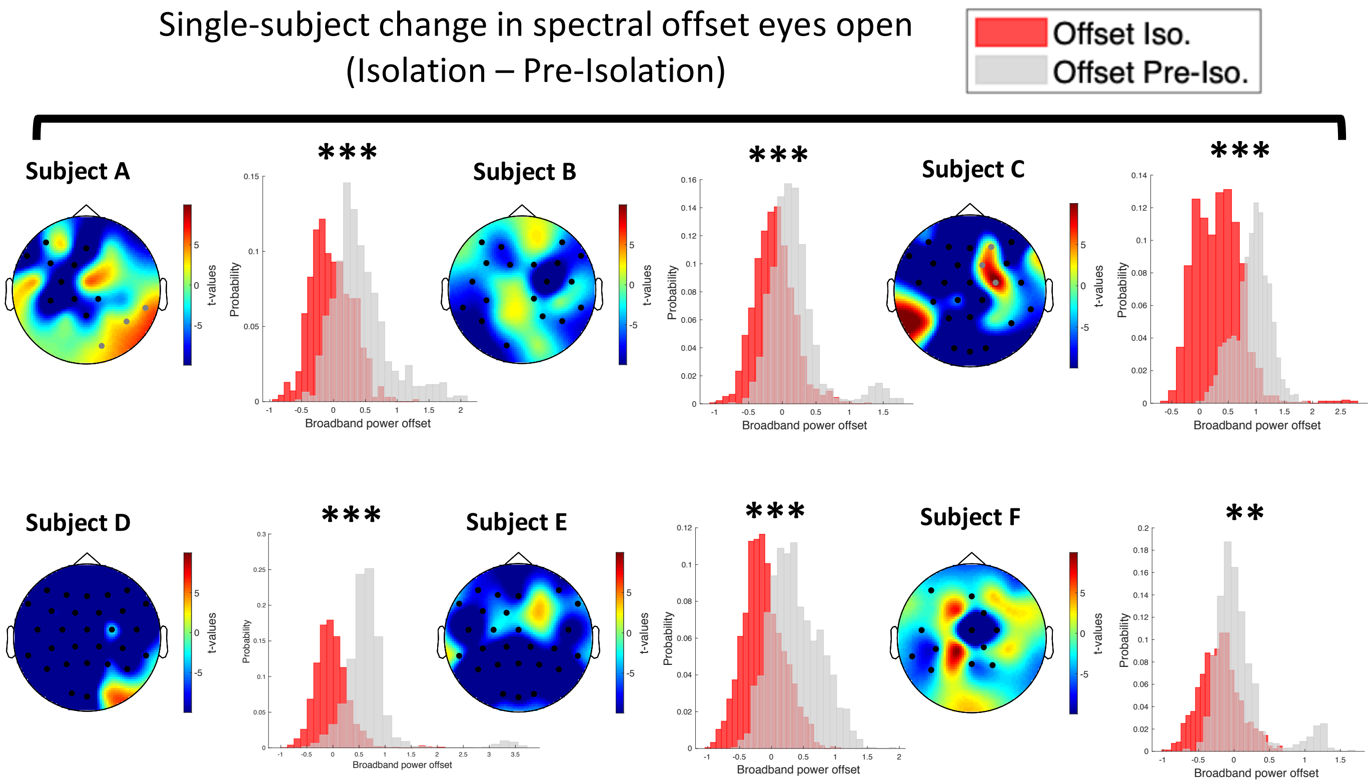

Single-subject change in spectral offset of the aperiodic signal from pre-isolation to isolation during eyes open condition. Histograms show the probability distribution of the spectral offset on a trial basis. Fitting of the aperiodic signal was performed on a trial basis using IRASA. The gray histogram reflects the probability distribution of the spectral offset prior to isolation. The red histogram reflects the probability distribution of the spectral offset during exposure to isolation. The topographical t-value distribution (obtained through random trial shuffling between the two conditions, *see* methods for details on single-subject cluster permutation analysis) obtained via cluster permutation is plotted for each condition difference (pairwise t-tests). Black dots represent significant sensors that are part of a negative cluster (*here:* spectral offset higher prior to isolation as compared to within isolation). Gray dots represent significant sensors that are part of a positive cluster (*here:* spectral offset lower prior to isolation as compared to within isolation). Individual subjects are denoted as *Subject A, B, C, D, E, F*. Note the consistent global reduction (6/6 subjects show a significant decrease) in spectral power offset from pre-isolation to isolation.
Legend: *** $p < 0.001$, ** $p < 0.01$

Figure S9: Change in spectral offset from isolation to post-isolation on a single-subject level for eyes open condition

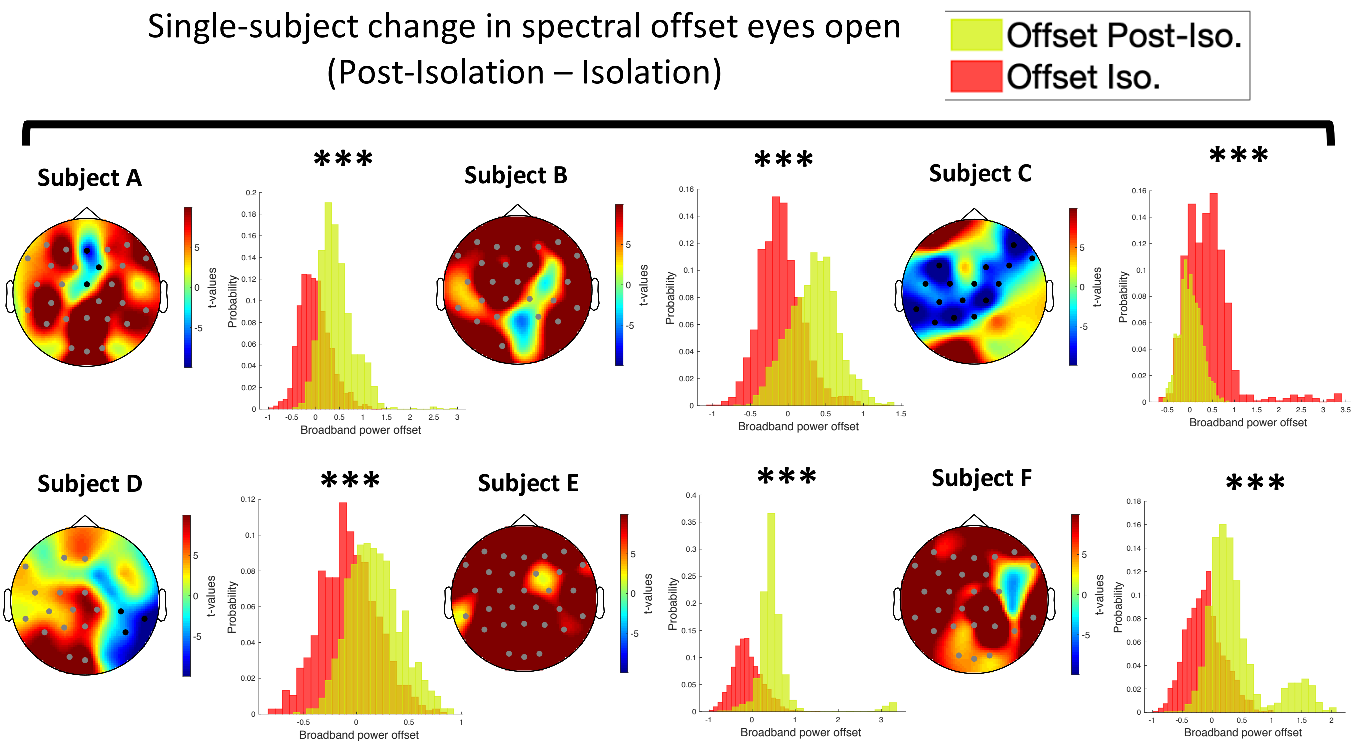

Single-subject change in spectral offset of the aperiodic signal from isolation to post-isolation during eyes open condition. Histograms show the probability distribution of the spectral offset on a trial basis. Fitting of the aperiodic signal was performed on a trial basis using IRASA. The red histogram reflects the probability distribution of the spectral offset during isolation. The green histogram reflects the probability distribution of the spectral offset post-isolation. The topographical t-value distribution (obtained through random trial shuffling between the two conditions, *see* methods for details on single-subject cluster permutation analysis) obtained via cluster permutation is plotted for each condition difference (pairwise t-tests). Black dots represent significant sensors that are part of a negative cluster (*here:* spectral offset higher during isolation as compared to post-isolation). Gray dots represent significant sensors that are part of a positive cluster (*here:* spectral offset lower during isolation as compared to post-isolation). Individual subjects are denoted as *Subject A, B, C, D, E, F*. Note that 5/6 subjects show an increase in spectral offset from isolation to post-isolation.
Legend: *** $p < 0.001$

Figure S10: Change in spectral slope from pre-isolation to isolation on a single-subject level for eyes open condition

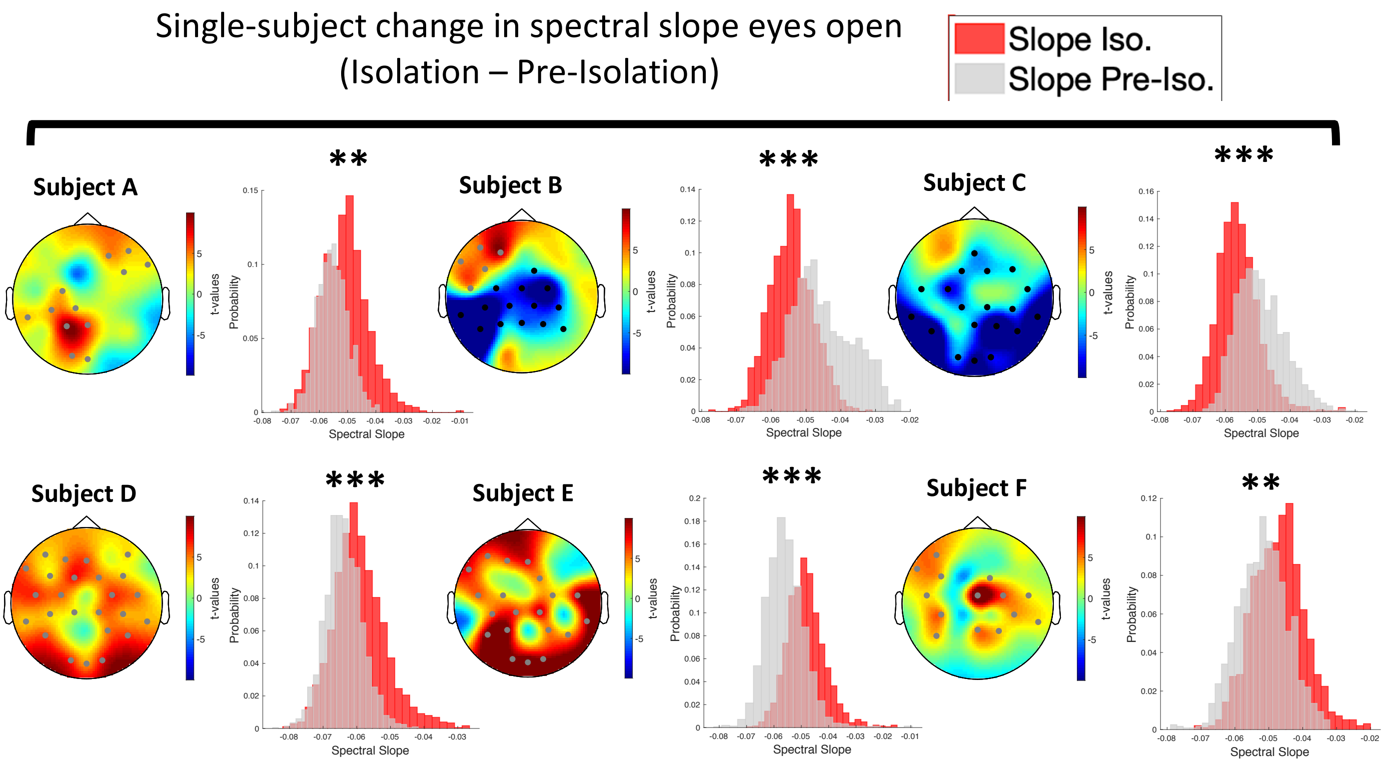

Single-subject change in spectral slope of the aperiodic signal from pre-isolation to isolation during eyes open condition. Histograms show the probability distribution of the spectral slope on a trial basis. Fitting of the aperiodic signal was performed on a trial basis using IRASA. The gray histogram reflects the probability distribution of the spectral slope prior to isolation. The red histogram reflects the probability distribution of the spectral slope during isolation. The topographical t-value distribution (obtained through random trial shuffling between the two conditions, *see* methods for details on single-subject cluster permutation analysis) obtained via cluster permutation is plotted for each condition difference (pairwise t-tests). Black dots represent significant sensors that are part of a negative cluster (*here:* spectral slope steeper during isolation as compared to pre-isolation). Gray dots represent significant sensors that are part of a positive cluster (*here:* spectral slope flatter during isolation as compared to pre-isolation). Individual subjects are denoted as *Subject A, B, C, D, E, F*. Note that 4/6 subjects show an increase in spectral slope from pre-isolation to isolation whereas 2/6 subjects show the opposite pattern.

Legend: *** $p < 0.001$, ** $p < 0.01$

Figure S11: Change in spectral slope from isolation to post-isolation on a single-subject level for eyes open condition

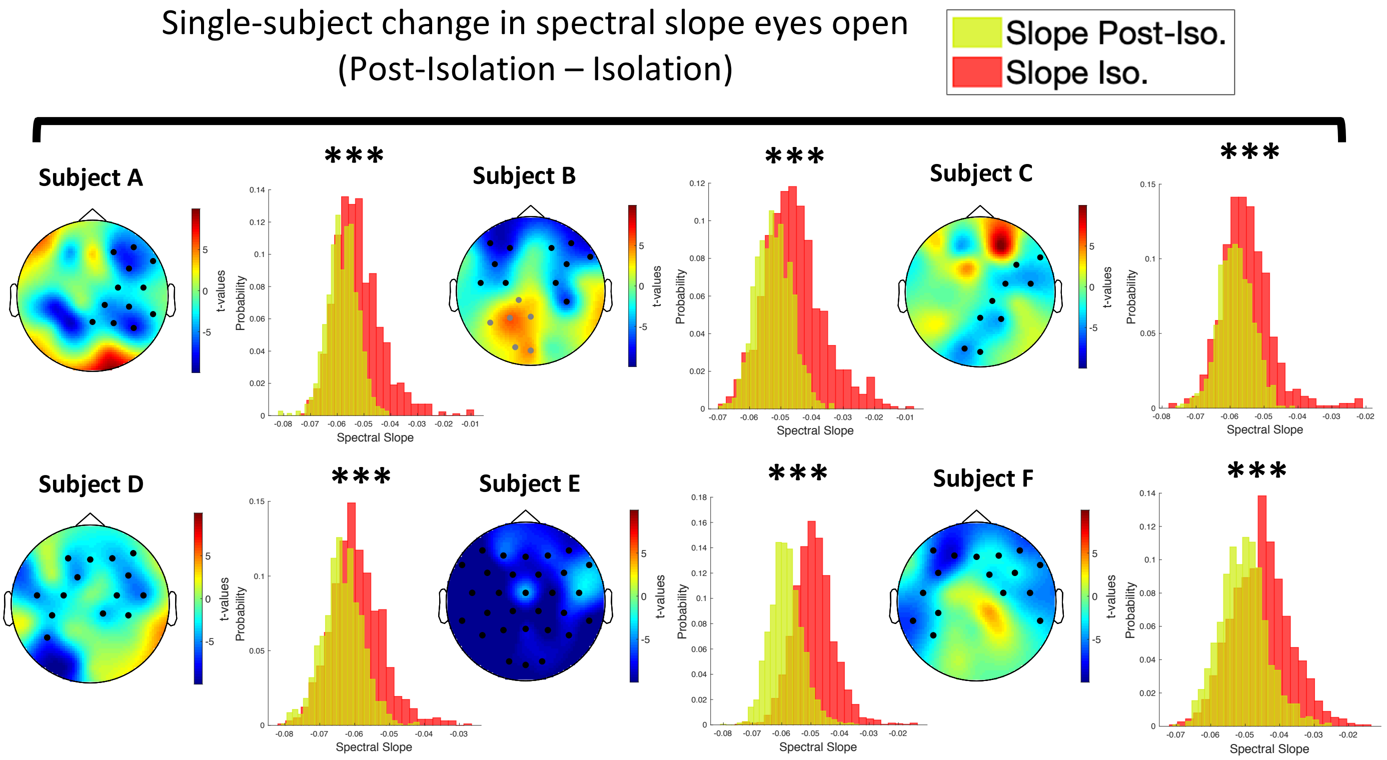

Single-subject change in spectral slope of the aperiodic signal from isolation to post-isolation during eyes open condition. Histograms show the probability distribution of the spectral slope on a trial basis. Fitting of the aperiodic signal was performed on a trial basis using IRASA. The red histogram reflects the probability distribution of the spectral slope during isolation. The green histogram reflects the probability distribution of the spectral slope post-isolation. The topographical t-value distribution (obtained through random trial shuffling between the two conditions, *see* methods for details on single-subject cluster permutation analysis) obtained via cluster permutation is plotted for each condition difference (pairwise t-tests). Black dots represent significant sensors that are part of a negative cluster (*here:* spectral slope flatter during isolation as compared to post-isolation). Individual subjects are denoted as *Subject A, B, C, D, E, F*. Note that 6/6 subjects show a decrease in spectral slope from isolation to post-isolation.

Legend: *** $p < 0.001$

Figure S12: Change in Alpha Peak Frequency from pre-isolation to isolation on a single-subject level for eyes closed condition

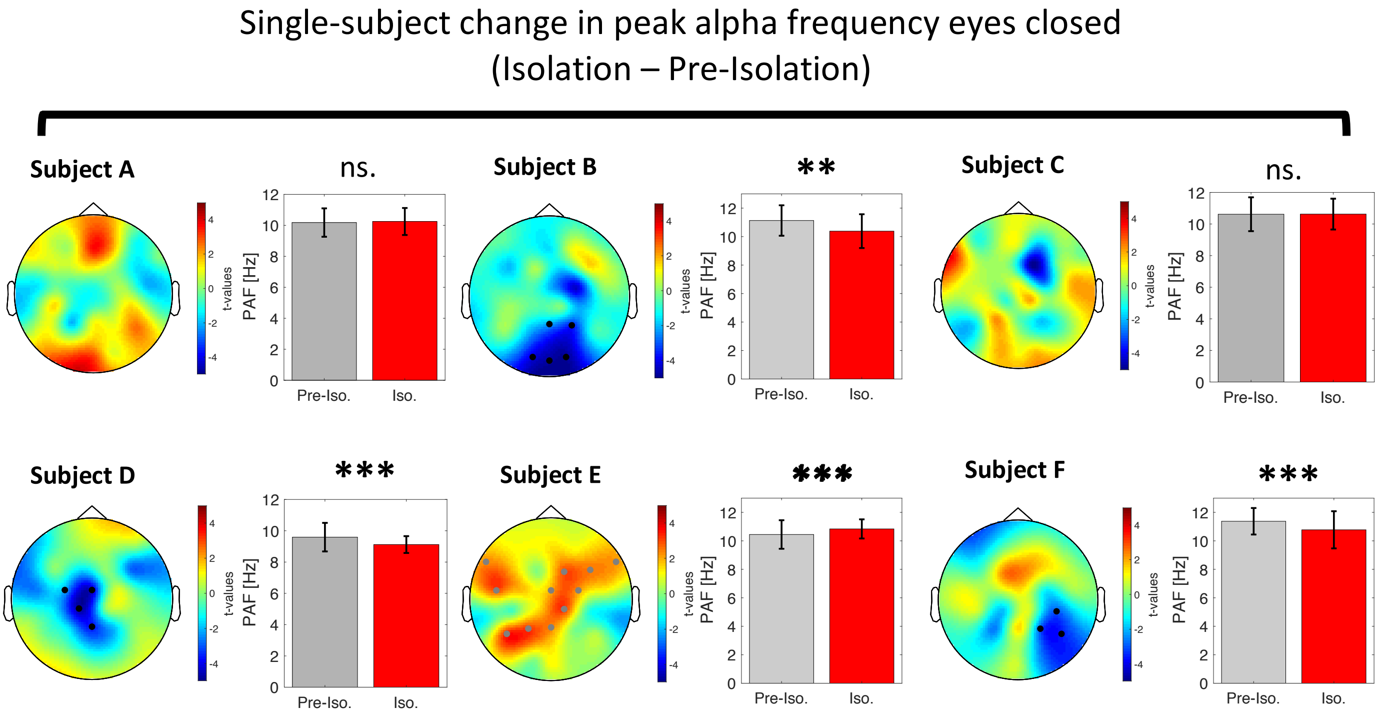

Single-subject change in Alpha Peak Frequency (APF) after removal of the aperiodic signal using IRASA. Bar plots show the mean APF during isolation (red) and post-isolation (green). Error bars represent the standard deviation over trials. The topographical t-value distribution (given by random trial shuffling between the two conditions, *see* methods for details on single-subject cluster permutation analysis) obtained via cluster permutation is plotted for each condition difference. Black dots represent significant sensors that are part of a negative cluster (*here:* APF reduced during isolation as compared to pre-isolation). Gray dots represent significant sensors that are part of a positive cluster (*here:* APF higher during isolation as compared to pre-isolation). Individual subjects are denoted as *Subject A, B, C, D, E, F*. Note that APF decreased in 3/6 subjects during isolation whereas 1 subject showed the opposite pattern and in 2 subject APF was not altered.
Legend: *** $p < 0.001$, ns = not significant

Figure S13: Correlation figures including alpha peak frequencies clustered around 9 Hz

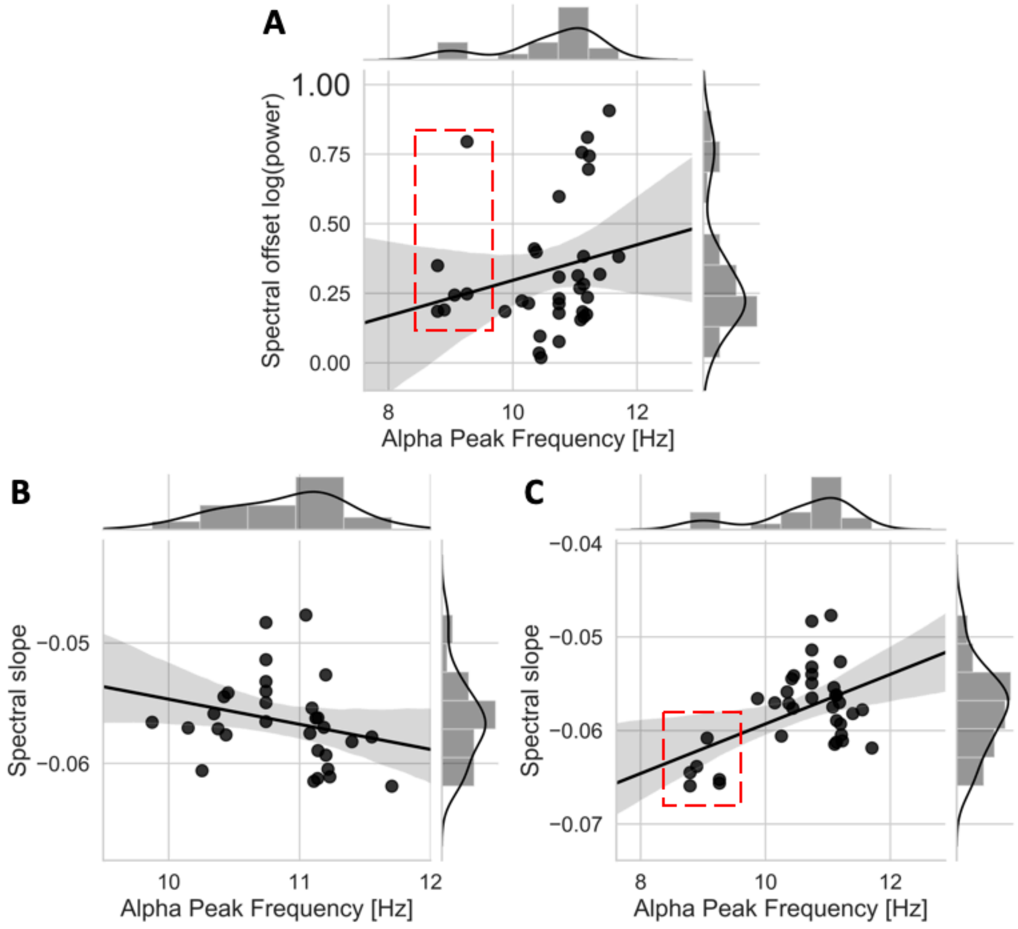

Pearson correlations between Alpha Peak Frequency and aperiodic features. (A) Correlation between Alpha Peak Frequency (APF, 1/f removed) and spectral offset of the aperiodic power spectrum. Note that the data clustered around 9 Hz (dashed box) is clearly out of the main cluster between approximately 10 – 12 Hz and leads to a non-normal distribution of the data (Shapiro-Wilk test including the clustered APF around 9 Hz: W(35) = 0.86, $p < 0.001$, one-tailed Spearman correlation (as data is non-normally distributed): r(34) = 0.29, $p = 0.046$; Shapiro-Wilk test without clustered APF around 9 Hz: W(29) = 0.95, $p = 0.17$, one-tailed Spearman correlation: r(28) = 0.437, $p = 0.007$ *[figure shown in main text]*). (B) Correlation between Alpha Peak Frequency (APF, 1/f removed) and spectral slope of the aperiodic power spectrum. Note that the APF data clustered around 9 Hz is removed here (Shapiro-Wilk test: W(35) = 0.86, $p < 0.001$, two-tailed Pearson correlation: r(28) = -0.26, $p = 0.17$). (C) Correlation between Alpha Peak Frequency (APF, 1/f removed) and spectral slope of the aperiodic power spectrum. Data includes the APF data clustered around 9 Hz (Shapiro-Wilk test: W(35) = 0.86, $p < 0.001$, two-tailed Spearman correlation: r(34) = 0.163, $p = 0.$ 34).

Figure S14: Control analyses to exclude possible confounds induced by variable daytime in EEG measurements

Due to complexity in the space-analogue environments and limited time capacity, it is not always possible to measure at the same time of the day. To exclude possible confounds induced by the daytime at which the measurement was taken, we performed several control analyses.

S14.1)

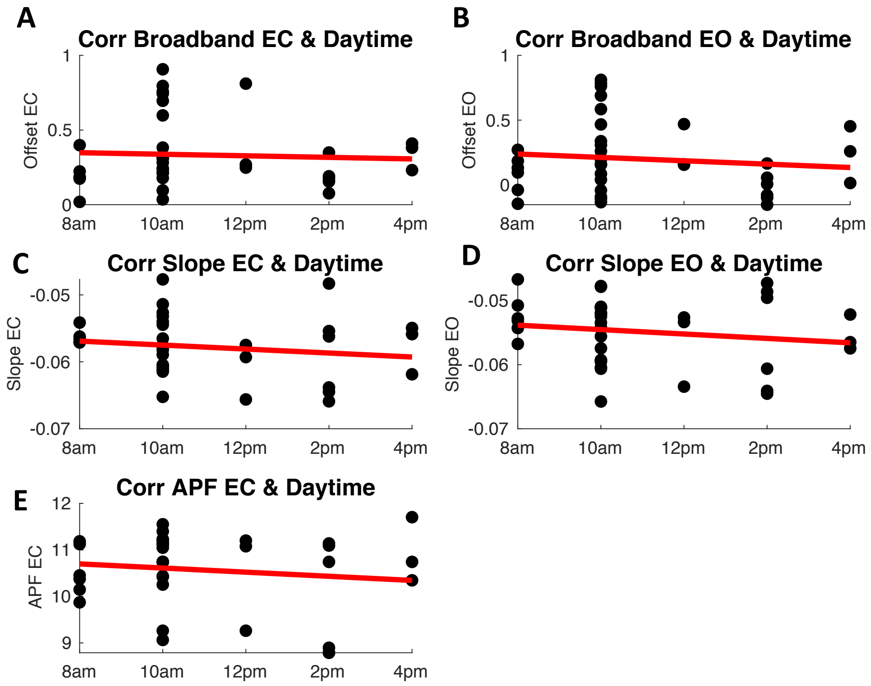

We binned the daytime into 5 bins between 8am to 6pm (8 – 10 am, 10 – 12noon, 12 – 2 pm, 2 – 4 pm, 4 – 6 pm) and adjusted the data accordingly to perform spearman correlations to examine whether there is a relationship between daytime and our dependent variables. We did not observe any significant correlation in any of our parameters.

(A) Spearman correlation between daytime and Broadband power offset during eyes closed: r = 0.07, p = 0.68

(B) Spearman correlation between daytime and Broadband power offset during eyes open: r = -0.07, p = 0.68

(C) Spearman correlation between daytime and spectral slope during eyes closed: r = -0.18, p = 0.29

(D) Spearman correlation between daytime and spectral slope during eyes open: r = -0.16, p = 0.36

(E) Spearman correlation between daytime and APF during eyes closed: r = -0.05, p = 0.79

S14.2)

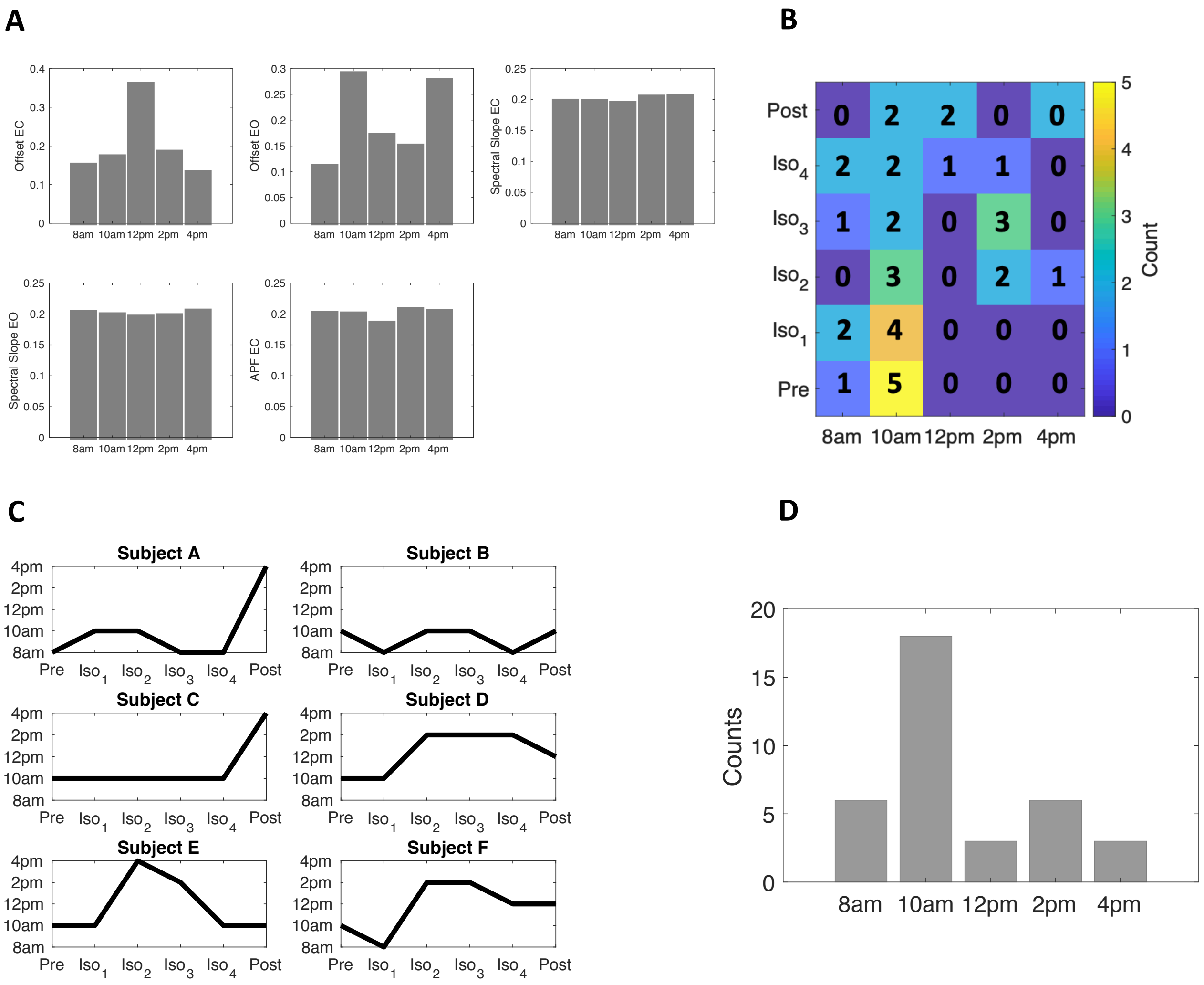

(A) Displays how the different parameters are modulated by daytime. Note that, except for the higher spectral offsets for measurements at 12pm during eyes closed and at 10am and 4pm during eyes open, no modulation by daytime is visible.

(B) The colormap displays the amount of measurements that have been taken at a respective daytime and isolation timepoints. Note that most of the measurements have been taken between 10 – 12 noon.

(C) Displays the daytime at which EEG has been recorded for each participant. Note that there is no systematic pattern which is likely to explain the consistent (across the majority of subjects as denoted in the single subject analyses) shift over time that we observed.

(D) Displays the number of EEG recordings that have been taken at a certain timepoint. Note that the majority of measurements has been taken between 10 – 12 noon.
